# Structural and biophysical insights into targeting of claudin-4 by a synthetic antibody fragment

**DOI:** 10.1038/s42003-024-06437-6

**Published:** 2024-06-17

**Authors:** Satchal K. Erramilli, Pawel K. Dominik, Chinemerem P. Ogbu, Anthony A. Kossiakoff, Alex J. Vecchio

**Affiliations:** 1 Department of Biochemistry and Molecular Biology, University of Chicago, Chicago, IL 60637 USA; 2 Department of Biochemistry, University of Nebraska-Lincoln, Lincoln, NE 68588 USA; 3 Present Address: Pfizer, San Diego, CA 92121 USA; 4 Present Address: Department of Structural Biology, University at Buffalo, Buffalo, NY 14203 USA

**Keywords:** Cryoelectron microscopy, Kinetics

## Abstract

Claudins are a 27-member family of ~25 kDa membrane proteins that integrate into tight junctions to form molecular barriers at the paracellular spaces between endothelial and epithelial cells. As the backbone of tight junction structure and function, claudins are attractive targets for modulating tissue permeability to deliver drugs or treat disease. However, structures of claudins are limited due to their small sizes and physicochemical properties—these traits also make therapy development a challenge. Here we report the development of a synthetic antibody fragment (sFab) that binds human claudin-4 and the determination of a high-resolution structure of it bound to claudin-4/enterotoxin complexes using cryogenic electron microscopy. Structural and biophysical results reveal this sFabs mechanism of select binding to human claudin-4 over other homologous claudins and establish the ability of sFabs to bind hard-to-target claudins to probe tight junction structure and function. The findings provide a framework for tight junction modulation by sFabs for tissue-selective therapies.

## Abstract (web summary)

A synthetic antibody fragment is engineered, capable of binding to human claudin-4, providing insights into its structure and selective binding mechanism, possibly enabling precise modulation of tight junctions in tissue-specific therapeutic interventions.

## Introduction

The claudin family of integral membrane proteins constitute the structural and functional backbone of endothelial and epithelial tight junctions in vertebrates. In mammals, 24+ claudin subtypes self-assemble, forming large homo- and/or heteromeric complexes that create barriers to the transport of small molecules and ions through intercellular spaces. Claudins have four transmembrane helices (TMs), which, along with two extracellular segments (ECS), enable tight junction assembly by simultaneously interacting laterally within the same membrane (cis) and across intercellular space with claudins on adjacent cell membranes (trans)^1,2^. Cis and trans interactions between the various sized and shaped claudin subtypes govern the frequency and morphology of tight junction strands, specifically tuning small molecule permeability to impart distinct molecular properties and physiological functions to tissues and organs^3^. Altering tight junction barrier permeability via transient disruption of claudin/claudin interactions is being investigated as a strategy to treat tight junction-linked diseases or to deliver drugs through these highly restrictive impediments, like across the blood-brain barrier^3–5^. Because trans interactions between claudin ECS domains direct paracellular barrier function, molecules that bind the ECS have a higher potential to alter molecular permeability. However, targeted binding of specific claudins with therapeutic agents, which would be required for tissue- or disease-specific treatments, remains a challenge.

Claudin-4 functions in normal physiological processes largely through its role in increasing the complexity of tight junctions and tuning the paracellular permselectivities of various tissues^6^. In lung epithelium, it regulates the paracellular barrier to modulate fluid clearance^7^. In kidney epithelium it is postulated to form an anion-selective channel vital for chloride reabsorption via interactions with claudin-8^8^. In intestinal epithelium, claudin-4 assists M cells, specialized cells that capture microbial microparticles and transports them through epithelium to immune cells, by acting as a structural component for M cell transcytosis vesicles^9^. Dysregulation of claudin-4 has been detected in bladder, breast, colorectal, gastric, lung, ovarian, pancreatic, prostate, and thyroid cancers^10,11^. Claudin-4 functions diversely in normal versus disease states, making it a prime target for therapeutics capable of modifying tight junction structure and function in claudin-4-expressing tissues.

In the gastrointestinal tracts of mammals, claudin-4 is also the primary endogenous receptor of *Clostridium perfringens* enterotoxin (CpE)^12,13^. CpE infection causes a highly prevalent form of food poisoning by disrupting tight junctions in the gut^14–16^. Claudin structural and sequence homology allows CpE to bind other subtypes with high affinity too^17,18^. We have previously pinpointed a conserved sequence that spans the ECS of claudins and found that it is recognized by CpE’s C-terminal domain (cCpE) to impart subtype-selective binding^19^. The N-terminal domain of CpE functions in cytotoxicity by structurally rearranging into a membrane-spanning β-barrel that induces cytotoxicity by creating a calcium-selective pore^15^. Although cCpE has no cytotoxic domain and does not kill cells, it is not harmless. Delivery of cCpE to the basolateral compartment of epithelial monolayers sequesters claudin-4 out of tight junctions, disrupting its barrier function^20^. The natural abilities of CpE and cCpE to bind claudins in a subtype-selective and high-affinity manner, while disrupting claudin barrier function through prevention of trans interactions, make them prospective modulating agents of tight junctions composed of CpE receptors like claudin-4. Specifically, CpE and cCpE are being used to target claudin-4 in ovarian, pancreatic, and other tissue-specific cancers^11,21–28^. Modified CpE and cCpE have been used to target claudins with low receptor capacities like claudin-5, to modulate opening of the blood-brain barrier^29–33^. Targeting of claudins by novel anti-claudin binders or by CpE enterotoxins thus represents a strategic and promising approach for the development of imaging probes, drugs, and therapeutics to treat tight junction-linked diseases.

Advancement of claudin binders is slow due to the small size, minute extracellular masses, physicochemical properties, homologous architectures, and dearth of experimentally derived structures of claudins—which could facilitate therapeutic efforts. To date, structures of two human and three murine claudins have been experimentally determined using X-ray crystallography to resolutions that illuminate their biology (<4.0 Å)^19,34–38^. The features that make claudins challenging to target with therapeutics also make them recalcitrant to structural determination—especially their proportionally low soluble ECS mass-to-hydrophobic TM ratios. Of the 27 human claudins, which range in size from 22-34 kDa, claudin-4 is the second smallest with 209 amino acids that amass to 22,077 Da. With an estimated 50% of claudin-4s mass being buried within membranes or disordered as termini, there is little “targetable” tertiary structure that presents itself as antigenic or binding surfaces for drugs. This small area is also all that is available to form the requisite protein/protein interactions for crystal nucleation. Detergents used to solubilize claudins for in vitro and structural studies additionally block protein interactions, crystallization, and drug-like molecules from binding claudins effectively. Developing approaches and/or molecules capable of circumventing the low mass-to-membrane ratios of claudins thus holds promise to augment both structural biology and tight junction modulating therapeutic efforts. Thus, the aim of this study was to develop a claudin-4-binding molecule to facilitate therapeutic efforts to target claudin-4-expressing tissues and use this molecule to pioneer new structural techniques capable of accelerating claudin structural biology.

We have developed a platform that synergizes structure determination and molecular targeting of claudins using synthetic antibody fragments (sFabs). By applying a sFab-encoding phage display library against detergent-solubilized human claudin-4 (hsCLDN-4) bound to cCpE, we have developed three sFabs termed CpE Obstructing Proteins (COPs). We have shown previously that COP-2 and COP-3 bind cCpE, but not hsCLDN-4, and used these COPs to determine low resolution (5–7 Å) structures of hsCLDN-4/cCpE complexes using single-particle cryogenic electron microscopy (cryo-EM)^39^. Here, we describe the development and function of COP-1, a hsCLDN-4-binding sFab, and employ it to reveal 2.2–2.6 Å structures of 22 kDa hsCLDN-4 bound to 14 kDa cCpE using cryo-EM—resolutions that exceed all claudin/cCpE structures determined previously with X-ray crystallography. Structural, biophysical, and biochemical analyses aid elucidation of the interactions required for CpE-induced cytotoxicity and COP-1s unique mechanism of hsCLDN-4-selective binding. This work represents the first high resolution structure of a claudin determined by cryo-EM and the discovery of COP-1 uncovers a potential new strategy to advance antibody-based therapies aimed at modulating tight junctions in tissue-specific ways through claudin-selective targeting.

## Results

### Development and sequence of COP-1

COP-1 was co-discovered with two anti-cCpE sFabs, using a phage display library encoding sFabs with variable complementarity-determining regions (CDRs) targeted against hsCLDN-4 solubilized in n-dodecyl-*β*-D-maltopyranoside (DDM) in complex with cCpE^39,40^. We sequenced COP-1 and compared it to the cCpE-binding sFabs COP-2 and -3, which revealed the expected variability in the CDR regions of the light (L) and heavy (H) chains (Supplementary Fig. 1). Sequence conservation is high in CDR-1 and CDR-2 of both L and H chains, with only minor alterations from Ser to Tyr between sFabs. However, the CDR-3 regions in both chains of each COP vary significantly. CDR-L3 of COP-1 has one aromatic residue compared to three and five in COP-2 and -3, and is truncated by one and two amino acids, respectively. COP-1s CDR-H3 has nine aromatic residues compared to seven and six in COP-2 and -3; there is also a one amino acid insert in COP-1, a Pro. This CDR-H3 Pro precedes four sequential Trp residues. We next expressed and purified COP-1 to characterize its ability to bind various antigens in vitro. We found that COP-1 was not as soluble as other COPs, which could be rationalized by its increased number of hydrophobic aromatic side chains in CDR-H3.

### Biochemical characterization of COP-1

**Fig. 1:**
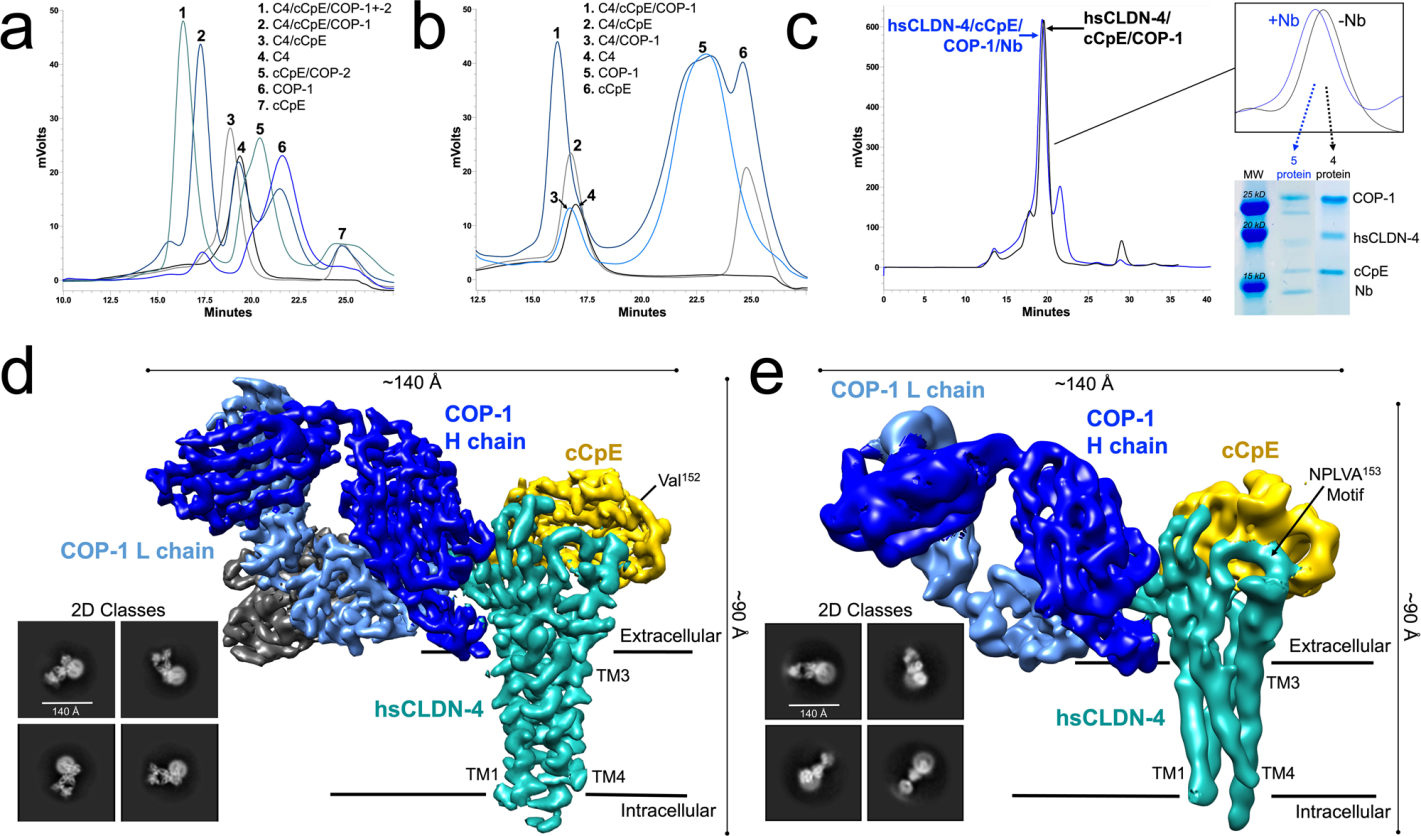
Biochemical Characterization, Preparation, and Structure Determination of Claudin-4/cCpE/COP-1 Complexes. **a** Analytical size-exclusion (SEC) chromatograms depicting elution profiles and peak retention times of various proteins and complexes between hsCLDN-4, cCpE, COP-1, and COP-2 in a mobile phase with DDM. **b** Analytical SEC chromatograms shown as in (**a**) but with a mobile phase containing DDM/CHS. Note that elution times of complexes in DDM/CHS are altered compared to (**a**) but that cCpE and COP-1 retention times are not. **c** Prep-scale SEC chromatograms of protein complexes used for cryo-EM with corresponding purity of the four- or five-protein complexes analyzed using SDS-PAGE. Note, these samples were prepared weeks apart and this image reflects the results from two gels, hence differences in mobility and intensity. **d** Cryo-EM overview of 2D classifications and 3D reconstruction of the hsCLDN-4/cCpE/COP-1/Nb complex 2.6 Å final map using a “loose” mask. The map is shown in relation to a model membrane boundary and the five proteins are colored accordingly: hsCLDN-4 (teal), cCpE (gold), COP-1 H (blue) and L (light blue) chains, and Nb (gray). Dimensions of the complex in 2D are provided. **e** Overview of 2D classifications and 3D reconstruction of the hsCLDN-4/cCpE/COP-1 complex 4.2 Å final map with the four proteins colored as in (**d**). Note that gross secondary structure is visible but not side chains at this resolution.

We biochemically characterized COP-1s ability to bind hsCLDN-4 and hsCLDN-4/cCpE complexes in vitro using size-exclusion chromatography (SEC) in DDM and DDM/cholesteryl hemisuccinate (CHS). The presence of CHS, a stabilizing molecule in membrane protein biochemistry, had no effect on binding results apart from earlier elution times of complexes due to increased mass caused by the incorporation of CHS into DDM micelles. Using this analysis, we showed that cCpE binds hsCLDN-4; COP-1 binds well to hsCLDN-4/cCpE complexes but only slightly to hsCLDN-4 alone; and COP-1 and COP-2 bind hsCLDN-4/cCpE complexes simultaneously (Fig. 1a, b). We had previously shown that COP-2 binds only to cCpE at a region opposite of hsCLDN-4^39^. Because COP-1 bound in the presence of COP-2, this suggested that COP-1s epitope was unique and that it may not bind cCpE. Moreover, the data showed that when hsCLDN-4/cCpE/COP-1 were complexed, excess COP-1 (peak 6) and cCpE (peak 7) did not result in a peak at the elution volume of cCpE/COP-2 complexes (peak 5), which indicated that COP-1 did not bind cCpE alone, unlike COP-2 (Fig. 1a). These analytical studies provided a foundation to probe COP-1 binding further using structural and biophysical techniques.

### Structure determination of hsCLDN-4/cCpE/COP-1 complexes by single-particle cryo-EM

We sought to determine the basis of COP-1 binding and to discern whether sFabs could yield high resolution structures of claudins using single-particle cryo-EM, which we postulated previously^39^. If so, cryo-EM could prove or disprove whether interactions hypothesized to be vital for hsCLDN-4 and cCpE association from ~3.5 Å crystal structures were present in an environment free from crystal-induced packing interactions^19,36^. Cryo-EM, therefore, had potential to provide new insights into claudin biology and expedite structural biology workflows.

HsCLDN-4 was purified in 2,2-didecylpropane-1,3-bis-β-D-maltopyranoside (LMNG) detergent, an analog of DDM but more cryo-EM amenable detergent, then bound to cCpE and COP-1 sequentially. This complex was used individually or also bound to an anti-sFab nanobody (Nb) known to bind to the hinge region between the variable and constant domains of sFab L chains^41^. We surmised that the Nb may help by adding mass to yield a > 100 kDa complex, by giving a unique shape to particles that could be readily distinguished in 2D classification, and by decreasing flexibility inherent to sFab constant domains. Both hsCLDN-4/cCpE/COP-1 and hsCLDN-4/cCpE/COP-1/Nb complexes were polished using SEC (Fig. 1c). These complexes were individually applied to EM grids, vitrified, and subjected to independent structural analyses using single-particle cryo-EM.

We collected a dataset containing 5039 movies of the hsCLDN-4/cCpE/COP-1/Nb complex first that yielded identifiable particles in 2D classification and 3D reconstructions (Fig. 1d). Initial processing generated a map that showed that all five proteins were present and was of sufficient quality to visualize protein secondary structural elements and side chains. Contouring this map revealed that density for the LMNG belt gave way to the four TM helix bundle intrinsic to the claudin fold. Further processing and refinement yielded a map that was resolved to a global resolution of 2.6 Å using a loose mask or 2.2 Å using a tight mask that minimized the micelle (Supplementary Fig. 2). Because the 2.6 Å refined map was sufficient to build and interpret a model, and the 2.2 Å map did not offer significantly improved map features, all subsequent structural interpretation employed the former map. As this structure was sufficient for structural interpretation, a smaller dataset of the hsCLDN-4/cCpE/COP-1 complex was collected and processed, resulting in a map with a final resolution of 4.2 Å that resolved the hole between COP-1 domains, the secondary structures and positions of all four proteins, as well as the four TMs and NPLVA^153^ motif of hsCLDN-4 (Fig. 1e and Supplementary Fig. 2)^19,42–44^. Map comparison showed no large difference apart from the inclusion of the Nb. We built structural models into both cryo-EM maps starting from density corresponding to the hsCLDN-4/cCpE complex using PDB ID 7KP4, then built COP-1 or COP-1/Nb manually. These models were then refined, constructing the final models of the hsCLDN-4/cCpE/COP-1/Nb and hsCLDN-4/cCpE/COP-1 complexes. Because the Nb did not alter COP-1 binding and this map was 1.5 Å higher resolution, all subsequent structural analyses focused exclusively on the 2.6 Å hsCLDN-4/cCpE/COP-1/Nb complex.

### Cryo-EM enables visualization of 22 kDa claudin-4

**Fig. 2:**
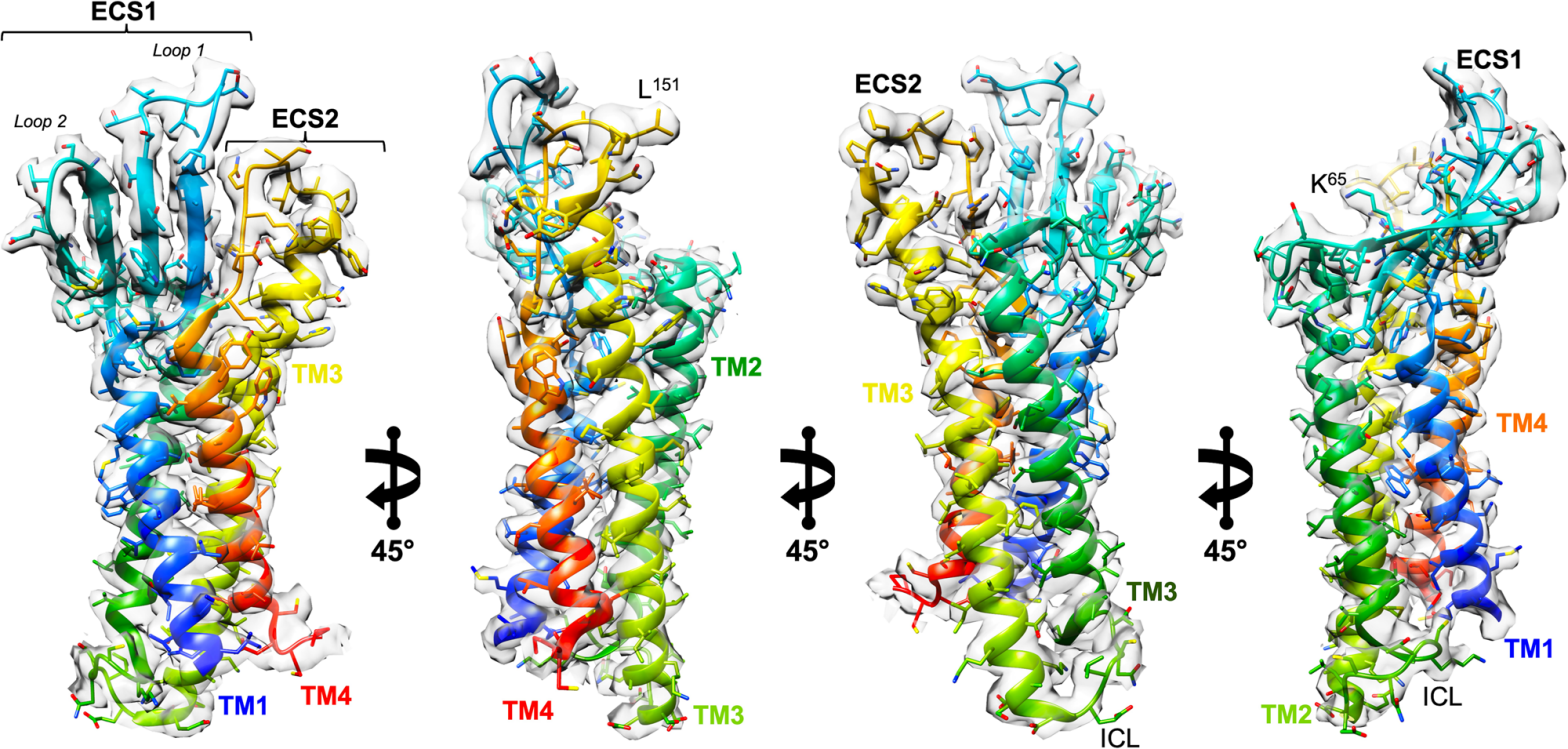
Visualization of 22 kDa Claudin-4. The 2.6 Å cryo-EM “loose” masked map from the hsCLDN-4/cCpE/COP-1/Nb complex (gray, translucent) overlaid on top of the structural model of hsCLDN-4 colored N- to C-terminus (blue to red). Carbons (teal), oxygens (red), nitrogens (blue), and sulfurs (yellow) are colored accordingly on side chains.

**Fig. 3:**
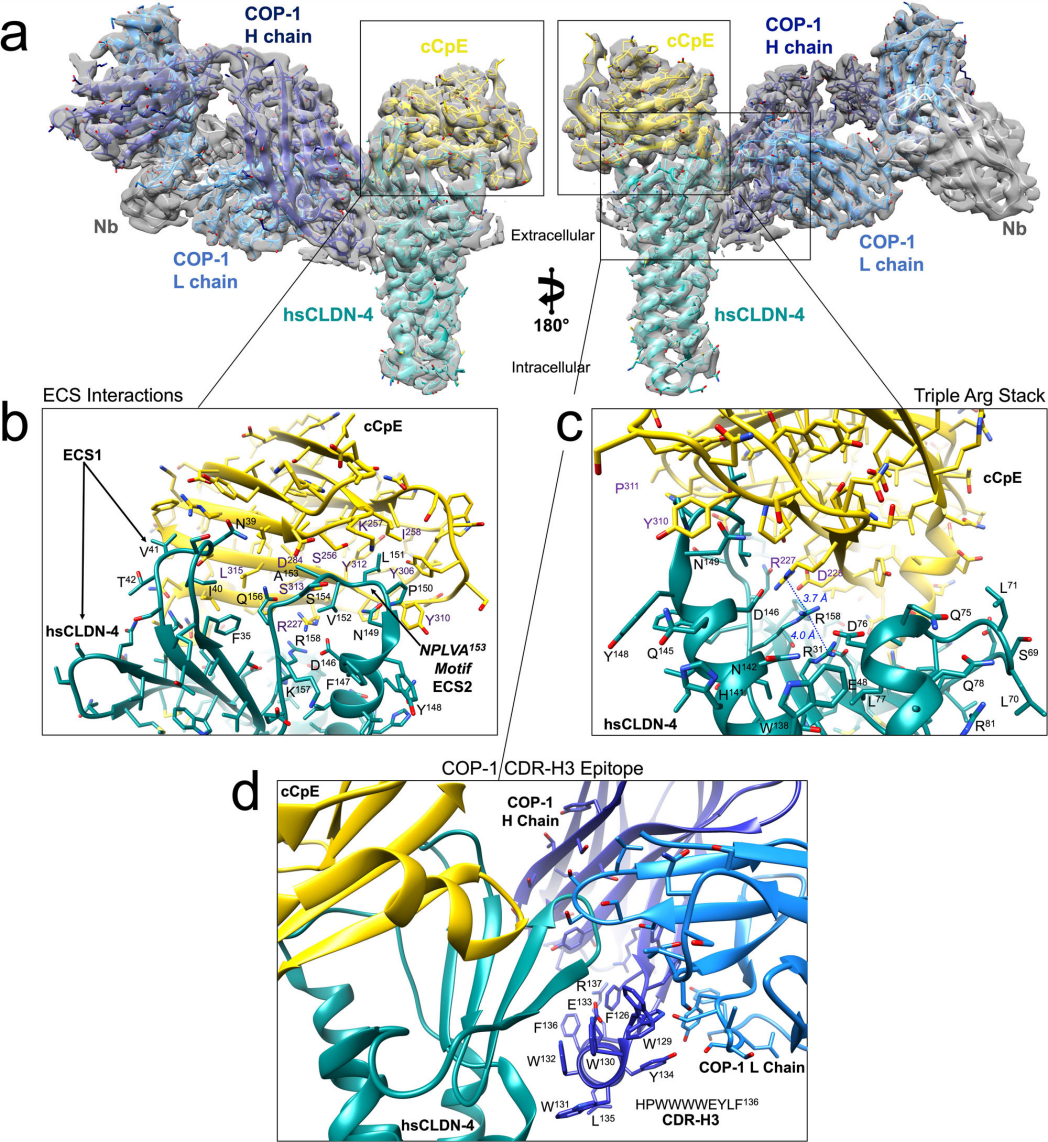
Structure of the Claudin-4/cCpE/COP-1/Nb Complex. **a** Global structure of the hsCLDN-4/cCpE/COP-1/Nb complex with hsCLDN-4 (teal), cCpE (gold), COP-1 (blue), and NB (gray) colored accordingly and overlaid map (gray). **b** Zoom-in of the ECS binding interface between hsCLDN-4 and cCpE. **c** Zoom-in of the interior binding interface between hsCLDN-4 and cCpE where three arginines form stacked interactions. **d** Zoom-in of the binding interface between hsCLDN-4 and COP-1, with the H chain (dark blue) and associated residues highlighted.

The utility of the 2.6 Å cryo-EM map became immediately apparent as we could visualize the complete tertiary structure of full-length hsCLDN-4 apart from its unstructured intracellular termini—180 of 209 residues (Fig. 2). This is significant because no experimentally determined claudin map had resolved all structural elements from TM1 to TM4, including a 2.4 Å crystal structure of mouse claudin-15^37^. The cryo-EM map clearly resolved all four TMs, both ECS and the single intracellular segment including their connecting loops and showed the positions of 180 side chains (Fig. 2 and Supplementary Fig. 3a, b). We performed local resolution analysis of the 2.6 Å map to approximate the resolution of different regions (Supplementary Fig. 3c). The average resolution of the map around hsCLDN-4 was <3.0 Å, with the intracellular loop connecting TM2 to TM3 exhibiting the most dynamics. Local resolutions of other regions showed that the variable domain of COP-1 and its antigen binding surfaces were resolved to <2.4 Å while the rest of the complex was resolved to between 2.6–3.0 Å. Importantly, the map resolved the structure and side chains of 14 kDa cCpE and its interface with hsCLDN-4 (Supplementary Fig. 3b). In full, the map was sufficient for precise placement of the entire hsCLDN-4/cCpE/COP-1/Nb complex within its density, which allowed for structure elucidation of cCpE binding to hsCLDN-4 and of COP-1s binding mode (Fig. 3a).

### Comparative analysis of cryo-EM and crystallographic structures of hsCLDN-4/cCpE

We first analyzed the hsCLDN-4/cCpE portion of the complex because the resolution of the cryo-EM map exceeded electron density maps produced by crystallography from two existing structures of the hsCLDN-4/cCpE complex (3.4 and 3.5 Å). This was undertaken to decipher how COP-1 binding and/or the lack of crystal packing interactions affected hsCLDN-4/cCpE interactions observed in crystal structures.

We structurally aligned the hsCLDN-4/cCpE portion of the complex from cryo-EM to the crystal structure of hsCLDN-4/cCpE (PDB ID 7KP4), which had a root mean square deviation of 0.90 Å between structures (Supplementary Fig. 4a). We found that global structural elements and side chain orientations are largely unchanged and interactions between hsCLDN-4 and cCpE were preserved. Specifically, the NPLVA^153^ motif, which imparts high-affinity cCpE binding to claudins (Fig. 3b); as well as the triple arginine stack at the hsCLDN-4/cCpE interface that imparts claudin-selective binding to cCpE (Fig. 3c), were both present in the cryo-EM structure^19,42–44^. The positions of these arginines are similar and Cζ-Cζ bond distances between hsCLDN-4 Arg158 and the other arginines were 3.7 and 4.0 Å from the cryo-EM structure, whereas in the crystal structure they were 3.5 and 3.8 Å^19^. This structural overlay also showed that two extracellular loops in ECS1 and the extracellular helix (ECH1) connecting *β*4–TM2 were conformationally different between structures (Supplementary Fig. 4b). The conformations of these regions found in the cryo-EM structure appeared to be altered due to COP-1 binding. The structure shows that CDR-L3 and CDR-H3 coordinate to sandwich the β3-β4 loop of hsCLDN-4 between them (Fig. 3d and Supplementary Fig. 4c). Although these COP-1-induced changes alter hsCLDN-4, they do little to alter the global orientation of cCpE or perturb critical interactions between it and hsCLDN-4. In fact, the structure shows that COP-1 does not directly interact with cCpE (Fig. 3a, d). Overall, the cryo-EM structure confirms the interactions and conformations hsCLDN-4 and cCpE utilize to engage in a high-affinity complex and validates the crystal structure, thus supporting conclusions made from that analysis.

### Deciphering hsCLDN-4’s extra-membraneous surface

**Fig. 4:**
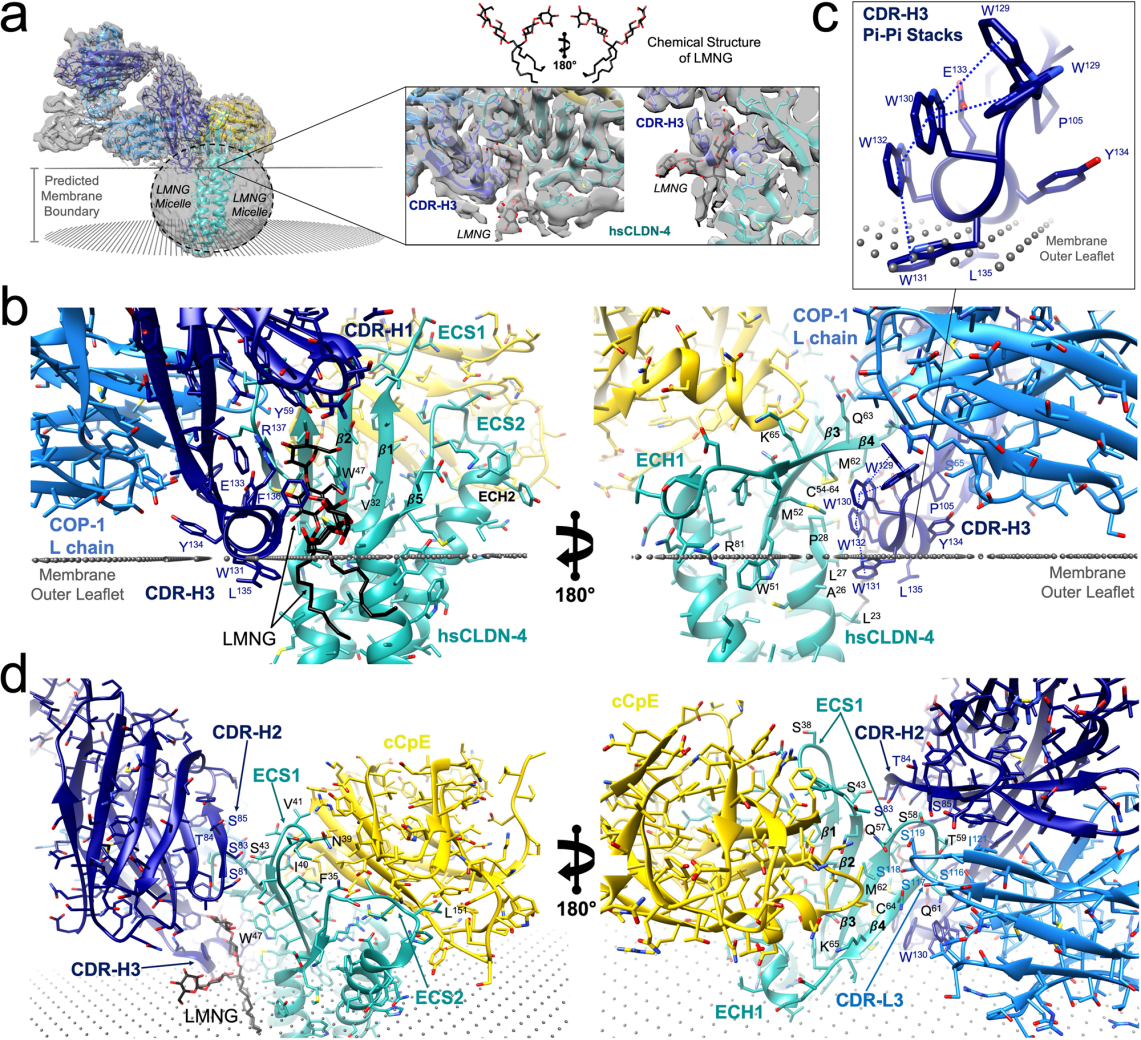
Structural Basis of COP-1 Recognition of Human Claudin-4. **a** The cryo-EM map from the hsCLDN-4/cCpE/COP-1/Nb complex (gray) contoured to show the experimentally determined boundary of the detergent belt with predicted membrane boundary from the PPM server (black dots)^45^ and structure overlayed for reference. Zoom-in of the interface between hsCLDN-4 (teal) and COP-1 (blue) showing density for a modeled LMNG detergent. The chemical structure of LMNG in a stick representation is also shown with carbons (black) and oxygens (red) colored accordingly. **b** Interactions between hsCLDN-4 and COP-1. The predicted PPM membrane boundary (black dots) overlaid on the structure of hsCLDN-4/cCpE/COP-1/Nb complex with hsCLDN-4 (teal), cCpE (gold), COP-1 (blue), and NB (gray), and LMNG colored accordingly as in (**a**). Side chains of significance are labeled and shown for reference. **c** Zoom-in on the structure of COP-1s CDR-H3 amphipathic helix showing π-π stacking and associated membrane penetration. **d** Interactions between COP-1s H (dark blue) and L (light blue) chains with the ECS1 region of hsCLDN-4.

Because detergent belts are resolvable in cryo-EM maps we could visualize what claudin surfaces are accessible to sFabs and infer the membrane boundaries (Fig. 4a). To validate whether the cryo-EM density for the detergent belt correlated to a logical membrane boundary, we input the structure into the Positioning Protein in Membranes (PPM) Server, a computational tool that energetically optimizes the spatial positions of membrane proteins in model membranes from structural coordinates^45^. PPM predicted a membrane bilayer that strongly correlated with the experimental density from the detergent belt, even though the belt was not included as input (Fig. 4a). The inner and outer leaflet boundaries resided at the extremities of the detergent belt torus. The predicted membrane was validated further by the presence of strong density between COP-1s CDR-H3 and hsCLDN-4, which we modeled as a LMNG detergent due to the density’s “X” shape, which fit LMNGs branched chain structure (Fig. 4a). The boundary of the outer membrane leaflet ended at the point between the sugar headgroup and acyl chain of LMNG, which was also not input into the server (Fig. 4b). The membrane boundary estimates thus made physicochemical sense and could be visually validated by the density for LMNG. This assessment showed that 96 (46%) of hsCLDN-4s 209 amino acids were membrane-embedded, leaving 113 (54%) to reside outside of the membrane and of those, 83 (40%) comprise a structured extracellular surface for molecular targeting. This analysis proved important because distinguishing the membrane boundary provided a marker to elucidate the basis of COP-1 binding to hsCLDN-4.

### Structural basis of COP-1 recognition of hsCLDN-4

COP-1s inability to significantly affect interactions between the hsCLDN-4/cCpE complex, we hypothesized, could be explained by its lack of interaction with cCpE. We therefore sought to decipher how COP-1 effectively targets and binds the complex using only hsCLDN-4, which has only a 40%-by-mass accessible antigenic surface. We analyzed COP-1s binding interface with hsCLDN-4 starting from the visualized detergent belt and estimated membrane boundary (Fig. 4b). The WWEYLF^136^ portion of COP-1s CDR-H3 forms an amphipathic α-helix that abuts and penetrates the detergent belt, interacting with both hsCLDN-4 and LMNG (Fig. 4b, c). Trp131 and Leu135 in the WWEYLF^136^ helix reside within the hydrophobic part of the detergent belt. Leu135 does not interact with hsCLDN-4 within the detergent belt but Trp131 does, forming non-polar interactions with Leu23, Ala26, and Leu27 on TM1. Other residues of the WWEYLF^136^ helix make interactions with hsCLDN-4 outside of the membrane. Trp130 and Trp132 form non-polar and polar interactions with hsCLDN-4; while Trp131, Trp132, Glu133, Leu135, and Phe136 also interact with LMNG (Fig. 4b). The helical structure of WWEYLF^136^ is facilitated by sequential π-π stacking interactions between Trp129 thru Trp132 in CDR-H3 where Trp129/130/132 form parallel-displaced and Trp131/132 T-shape stacks (Fig. 4c). The density for Trp129 showed that it has an alternate conformation that can parallel-displace or T-shape stack with Trp130. Trp129 dynamics may be due to Ser55 of CDR-L1, which resides 3.2–3.7 Å away, within hydrogen bonding distance. Sequence alignments show that the PWWWWEYLF^136^ sequence of COP-1s CDR-H3 is not present in other COPs (Supplementary Fig. 1). COP-1s ability to penetrate a hydrophobic environment gives it access to generally inaccessible TM regions, effectively increasing the accessible surface area on hsCLDN-4 for targeting.

Other regions and residues of COP-1 outside of CDR-H3 also form interactions of significance. Tyr59 and His61 of CDR-H1 interact with the β3-β4 loop of hsCLDN-4 ECS1. In CDR-H2, Ser83 and 85 interact with both ECS1 extracellular loops—the former with Ser43 of hsCLDN-4 (Fig. 4d). In full, eight residues of COP-1s H chain interact with 12 side chains on three epitopes of hsCLDN-4 (Supplementary Table 1). Epitope 1 constitutes residues LCCALPM^29^; epitope 2, TSQTIW^46^; and epitope 3, QSTGQMQC^47^ of hsCLDN-4 (Supplementary Fig. 5a). COP-1s L chain plays a less prominent role than chain H with only six side chains making interactions with five side chains on epitope 3 of hsCLDN-4 (Supplementary Table 1). The two COP-1 chains overlap interaction sites on hsCLDN-4 as both bind epitope 3 (Supplementary Fig. 5b, c). In chain L, CDR-L3 drives most interactions with Ser 116, 117, and 118 forming up to 10 potential hydrogen bonds with hsCLDN-4 epitope 3 residues Gln57, Ser58, Thr59, Gln61, and Gln63 (Fig. 4d). In sum, COP-1 employs both chains and five of six CDRs to bind distinct portions of hsCLDN-4 that reside inside and outside of the membrane. We hypothesized that COP-1s membrane penetration could drive binding to hsCLDN-4.

### COP-1 binds claudins in absence of enterotoxin

We tested whether COP-1 bound hsCLDN-4 selectively based on interactions observed in the cryo-EM structure by determining the affinity and kinetics of its interactions to it and other subtypes. Using bio-layer interferometry (BLI), we established qualitative and quantitative binding analyses between claudins, cCpE, CpE, and COP-1 (Supplementary Fig. 6). We first validated cCpE binding to human claudins -3 (hsCLDN-3), -4, and -9 (hsCLDN-9); and mouse claudins -3 (mmCLDN-3) and -4 (mmCLDN-4). These claudins were chosen due to their known receptor capacities for CpE and sequence conservation (Supplementary Fig. 7). We determined rates for the second-order association (k_on_) and first-order dissociation (k_off_) constants, and equilibrium dissociation constant (K_D_), and found that all claudins except hsCLDN-3 bound with <12 nM affinities (Table 1 and Supplementary Fig. 6a–e and Supplementary Table 2). Although the affinity of cCpE to mmCLDN-4 has not been previously reported, all other values agreed with our previously published values^38,48^. These results initiated in-depth analyses of COP-1 binding to claudin/enterotoxin complexes.

**Table 1:** Binding of Claudins to cCpE and COP-1 to Claudin/cCpE Complexes

Subtype	cCpE Binding		Subtype/cCpE	COP-1 Binding		
	*K*_D_ (nM)	*t*_1/2_ (min)		*K*_D1_ (nM)	*K*_D2_ (µM)	*t*_1/2_ (min)
hsCLDN-3	374.7 ± 2.1	5.0	hsCLDN-3/cCpE	339.9 ± 9.4	0.7 ± 1.5	0.1
hsCLDN-4	7.7 ± 0.1	55.0	hsCLDN-4/cCpE	23.4 ± 1.7	8.2 ± 6.6	21.2
hsCLDN-9	4.9 ± 0.1	88.9	hsCLDN-9/cCpE	120.6 ± 0.8	1.4 ± 0.1	2.4
mmCLDN-3	9.0 ± 0.1	25.7	mmCLDN-3/cCpE	47.3 ± 1.0	42.0 ± 24.9	10.6
mmCLDN-4	11.9 ± 0.1	14.1	mmCLDN-4/cCpE	60.5 ± 2.1	22.2 ± 4.0	11.9

BLI was used to measure binding kinetics and affinities. Binding of claudins to cCpE represent a single experiment and was fit with a 1:1 binding model, while COP-1 binding measurements represent averages from duplicate experiments and were fit with a 2:1 heterogenous ligand model, hence two *K*_Ds_ are reported. The t_1/2_ (ln 2/k_off_) was calculated from the dissociation rates between claudin/cCpE and COP-1 that represent *K*_D1_. The full binding results, including kinetic rates, appear in Supplementary Table 2.

**Fig. 5:**
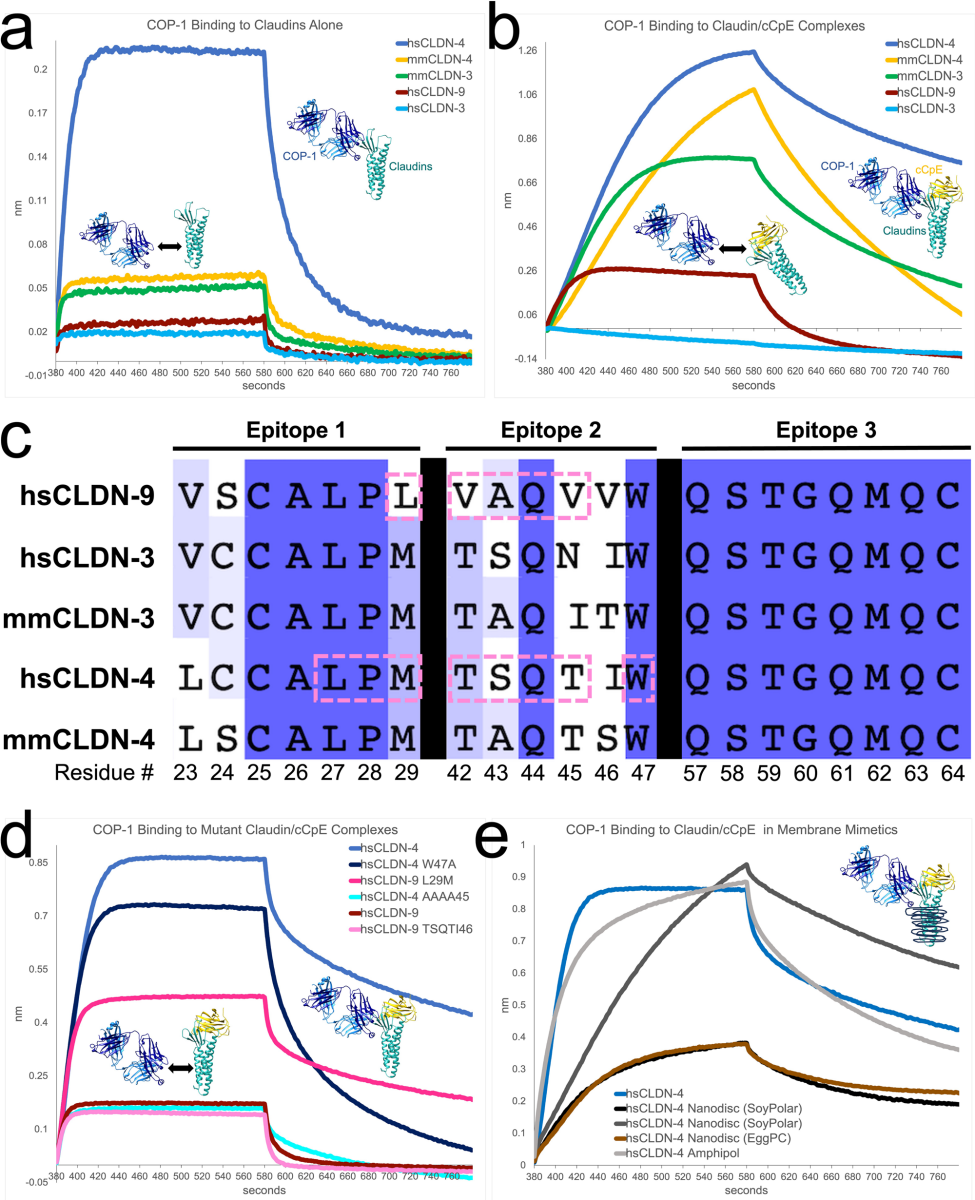
Biophysical Characterization of COP-1 Binding. **a** COP-1 binds to claudins in the absence of enterotoxins. COP-1 binding is maintained best with hsCLDN-4 but weaker with other subtypes. **b** COP-1 binding to claudin/cCpE complexes shows improved binding capacity compared to claudin alone for most subtypes assayed; hsCLDN-3 (light blue trace) is an exception. Note that the hierarchy of best binding subtypes is similar to claudins in absence of cCpE. **c** Sequence alignment of homologous claudins in their COP-1 binding epitopes. Because epitope 3 was 100% conserved, regions in epitopes 1 and 2 were mutated in hsCLDN-4 and -9 (pink dashed boxes) to validate structures. **d** COP-1 binding to mutant claudin/cCpE complexes shows that all hsCLDN-4 mutants lose binding capacity for COP-1, while for hsCLDN-9, mutations to epitope 2 (pink trace) lose binding while a Leu29Met point mutant in epitope 1 (dark pink trace) gains binding capacity to near wild type hsCLDN-4 (blue trace) levels. **e** Wild type hsCLDN-4 was reconstituted in synthetic amphipol polymers or lipid nanodiscs to test COP-1 binding in non-detergent systems. Results show that COP-1 binds to hsCLDN-4/cCpE complexes in each membrane mimetic but the kinetics of binding are altered.

We next qualitatively assessed claudin binding to CpE; COP-1 binding to cCpE; and COP-1 binding to claudins using single-point analyses (Supplementary Table 3). We found that all claudins bound CpE with a hierarchy of affinities of hsCLDN-4>hsCLDN-9>mmCLDN-3>mmCLDN-4»hsCLDN-3 (Supplementary Fig. 6f). Because k_on_ and k_off_ rates were measured, we fit the single-point binding curves to generate approximate *K*_Ds_. These results mirrored those from binding to cCpE, with hsCLDN-3 binding more weakly than other subtypes. We then determined that COP-1 did not bind to cCpE (Supplementary Fig. 6g). This result verified our biochemical assay where we found that excess COP-1 and cCpE did not elute as a complex (Fig. 1a). Lastly, we found that COP-1 bound to claudins in absence of cCpE, exhibiting selectivity for hsCLDN-4 (Fig. 5a). As before, we fit the single-point binding curves to a binding model to generate approximate *K*_Ds_ (Supplementary Fig. 6h). But unlike claudin binding to enterotoxins, which fits a 1:1 model, COP-1 binding data fit best to a 2:1 heterogeneous ligand model. This binding K_D_ analysis revealed a hierarchy of hsCLDN-4>mmCLDN-4>hsCLDN-3>mmCLDN-3>hsCLDN-9 for COP-1 binding to claudins. These analyses showed that COP-1 binds to claudins but not cCpE, so we next determined COP-1 binding to claudin/enterotoxin complexes as this knowledge was relevant to understand the structures.

### COP-1 selectively binds hsCLDN-4/cCpE complexes over other subtypes

To qualitatively grasp COP-1 binding to claudin/enterotoxin complexes we performed single-point analyses (Supplementary Table 3). Here, pre-formed claudin/cCpE complexes were immobilized then binding was tested against COP-1. Results showed that COP-1 bound claudin/cCpE complexes with specificity for hsCLDN-4/cCpE>mmCLDN-3/cCpE>mmCLDN-4/cCpE>>hsCLDN-9/cCpE—COP-1 did not bind hsCLDN-3/cCpE (Fig. 5b and Supplementary Fig. 6i). We then tested COP-1 binding to claudin/CpE complexes, as full-length CpE contains both N-terminal and cCpE domains. We found that COP-1 bound with identical preferences to claudin/CpE complexes as to claudin/cCpE—again COP-1 failed to bind hsCLDN-3/CpE (Supplementary Fig. 6j). K_Ds_ were estimated using a 2:1 model for COP-1 binding to both claudin/enterotoxin complexes, which resulted in the aforementioned preferences in binding. These results showed that COP-1 was selective for hsCLDN-4/enterotoxin complexes despite sequence identities between subtypes that ranged from 65.7% for hsCLDN-9, 68.3% for mmCLDN-3, 69.2% for hsCLDN-3, to 83.7% for mmCLDN-4 (Supplementary Fig. 7).

We next conducted complete quantitative studies with claudin/cCpE to determine the kinetics and affinities of COP-1 binding to comprehend our structures in a better light (Supplementary Table 2). Using pre-formed claudin/cCpE complexes we tested binding against six concentrations of COP-1 to hsCLDN-3/cCpE (Supplementary Fig. 6k), hsCLDN-4/cCpE (Supplementary Fig. 6l, p), hsCLDN-9/cCpE (Supplementary Fig. 6m), mmCLDN-3/cCpE (Supplementary Fig. 6n), and mmCLDN-4/cCpE (Supplementary Fig. 6o). This data also fit best to a 2:1 heterogeneous ligand binding model and thus two K_Ds_ are reported, one for each of the estimated two COP-1 interactions. We found that COP-1 bound with an average K_D1_ of 339.9, 23.4, 120.6, 47.3, and 60.5 nM (Table 1 and Supplementary Table 2). The hierarchy of COP-1 affinity was hsCLDN-4/cCpE>mmCLDN-3/cCpE>mmCLDN-4/cCpE>hsCLDN-9/cCpE>hsCLDN-3/cCpE, which was in accordance with the single-point results (Supplementary Fig. 6i). In this experiment, COP-1 binding to hsCLDN-3/cCpE was detected because higher concentrations were used. We used the k_off_ rates to calculate the half-life (t_1/2_) of claudin/cCpE/COP-1 complexes because this parameter provides a time component that expands understanding of complex binding over affinity measurements alone. The hierarchy of calculated COP-1 half-lives were hsCLDN-4/cCpE>mmCLDN-4/cCpE>mmCLDN-3/cCpE>hsCLDN-9/cCpE>hsCLDN-3/cCpE—mmCLDN-4 and -3 changed positions in the hierarchy using this parameter (Table 1). COP-1 remained bound to claudin-4 subtypes longer than others. These findings provided more precise measurements by which to elucidate the biophysical and mechanistic bases of COP-1s targeting and selective binding to claudins with high homologies.

### Biochemical validation of COP-1 selectivity

We next determined whether COP-1-bound claudin/cCpE complexes could be retained through SEC to validate the biophysical findings. Here, pre-formed claudin/cCpE complexes were removed from wells used for BLI then injected onto a column and analyzed using SEC. Another aliquot of these same complexes was incubated with COP-1 then injected on SEC. Results show that in the absence of COP-1, all claudin/cCpE samples elute between 10.25–10.60 min (Supplementary Fig. 8a). When COP-1 is added, larger molecular weight complexes were formed by hsCLDN-4/cCpE, mmCLDN-3/cCpE, and mmCLDN-4/cCpE, as determined by decreased peak elution times <10 min (Supplementary Fig. 8b). For hsCLDN-9/cCpE, two peaks eluted, one <10 min and another at 10.45 min. For hsCLDN-3/cCpE, the peak elution time with COP-1 was 10.40 min, similar to 10.60 min without COP-1. To validate that the peaks eluting at <10 min had COP-1-bound, we incubated hsCLDN-4/cCpE with COP-2, a cCpE-binding sFab, and found that this complex eluted at 9.50 min (Supplementary Fig. 8b). In full, this experiment showed that COP-1 retained complexes with hsCLDN-4, mmCLDN-3, and mmCLDN-4, while hsCLDN-9 formed a partial complex and hsCLDN-3 did not bind COP-1 (Supplementary Fig. 8c). These findings validated our biophysical results by showing which claudin/cCpE complexes bind COP-1 with high affinity.

### Claudin mutants at COP-1 epitopes alter binding

We next aimed to determine how mutations to claudins affected COP-1 binding to validate the amassed structure/function findings. Based on sequence homology, we mutated residues in epitopes 1 and 2 of hsCLDN-4 and hsCLDN-9, the best and worst COP-1 binders respectively, with the intent of disabling COP-1 binding to hsCLDN-4 and improve its binding to hsCLDN-9 (Fig. 5c). We did not alter epitope 3 because its sequence is 100% conserved in the claudins used in this study and was thus unlikely a determinant of subtype-specific COP-1 binding. We made three mutants of hsCLDN-4 that included hsCLDN-4^LPM-AAA29^ on epitope 1, and hsCLDN-4^W47A^ and hsCLDN-4^TSQT-AAAA45^ on epitope 2, and two mutants of hsCLDN-9 that included hsCLDN-9^L29M^ on epitope 1 and hsCLDN-9^VAQVV-TSQTI46^ on epitope 2 (Fig. 5c). The latter mutant swapped the sequence of hsCLDN-9 with that of hsCLDN-4. All mutants were then expressed and purified in DDM using identical methods to wild type proteins. Unfortunately, we found that the hsCLDN-4^LPM-AAA29^ mutant was insoluble and not able to be assessed. However, all others were further analyzed.

We tested cCpE and COP-1 binding to mutant claudins using single-point qualitative analyses (Supplementary Table 3). We first tested cCpE binding to mutant claudins to discern if mutations affect cCpE binding in absence of COP-1 (Supplementary Fig. 9a). Results showed that all claudin mutants except hsCLDN-4^TSQT-AAAA45^ bound cCpE with wild type-like trends. The k_off_ of this mutant was faster than wild type hsCLDN-4, resulting in a 7-fold lower K_D_. We next made pre-formed mutant claudin/cCpE complexes and tested COP-1 binding (Supplementary Fig. 9b). We found that the mutant claudin/cCpE complexes bound COP-1 to various degrees. Specifically, the hsCLDN-4^W47A^, hsCLDN-4^TSQT-AAAA45^, and hsCLDN-9^VAQVV-TSQTI46^ mutants bound COP-1 less well than wild type hsCLDN-4 or -9; while the mutant hsCLDN-9^L29M^ exhibited improved binding to COP-1 compared to wild type hsCLDN-9 (Fig. 5d). In fact, the hsCLDN-9^L29M^ mutant binding resembled wild type hsCLDN-4 more than hsCLDN-9. The results showed that the hierarchy of COP-1 binding was hsCLDN-4>hsCLDN-9^L29M^>hsCLDN-4^W47A^>hsCLDN-4^TSQT-AAAA45^>hsCLDN-9>hsCLDN-9^VAQVV-TSQTI46^. These results revealed the influence of claudin epitopes 1 and 2 in COP-1 subtype-specific binding.

### COP-1 binds claudins in membrane mimetics

Because we observed LMNG detergent in the cryo-EM structure bound between COP-1s CDR-H3 and hsCLDN-4, we wanted to discern whether COP-1 binding was influenced by detergents and assess if COP-1 could bind claudins in vitro or in vivo for varied applications. We exchanged DDM-solublized hsCLDN-4 into a synthetic amphipathic polymer called amphipol A8-35 and lipoprotein nanodiscs, two membrane mimetics used for in vitro analysis of membrane proteins in the absence of detergents and that are structurally distinct from maltoside detergents^46,49^. We first tested hsCLDN-4 in amphipols and found that the magnitude of cCpE binding was less than hsCLDN-4 in DDM (Supplementary Fig. 9c). We then pre-formed hsCLDN-4/cCpE complexes in amphipol and found that COP-1 binding was approximately as robust as it was to complexes in detergent, although the k_on_ and k_off_ rates differed (Fig. 5e and Supplementary Fig. 9d). We then reconstituted hsCLDN-4 into nanodiscs composed of lipids from two different sources, soybean polar lipid extracts (soy polar) or L-α-phosphatidylcholine isolated from chicken egg (eggPC), and tested binding to cCpE. This revealed that cCpE bound equivalently to hsCLDN-4 in nanodiscs as it does in detergent and that cCpE binding was concentration-dependent (Supplementary Fig. 9e). We pre-formed hsCLDN-4 nanodisc/cCpE complexes and found that COP-1 bound to them in a concentration-dependent way and that measured K_Ds_ were 2.2–4.1-fold lower in lipids than in DDM (Fig. 5e). This affinity difference appears a result of slower k_on_ rates of COP-1 in nanodiscs. The results of these binding studies showed that COP-1 binds hsCLDN-4 best in DDM but that it also binds with <100 nM affinity to hsCLDN-4 in synthetic or lipid-based membrane mimetics (Supplementary Fig. 9f). Coupled with our structural, biophysical, and biochemical results, these findings reveal COP-1s basis for subtype-selectivity and provide a framework to use sFabs to bolster structural and therapeutic efforts that target claudins.

## Discussion

We have developed a platform using hsCLDN-4/cCpE complexes and a phage display library encoding sFabs that yields selective binding molecules and molecular structures of claudins. We previously showed that the cCpE-binding sFabs COP-2 and COP-3 enable low resolution structures of claudins by cryo-EM^39^. Now, using the claudin-binding sFab COP-1 we demonstrate that COPs enable high resolution structure determination of 22 kDa claudin-4 and its complex with a 14 kDa enterotoxin fragment from *Clostridium perfringens* with side chain-level precision using cryo-EM. The 2.6 Å resolution achieved using this platform surpasses X-ray crystal structures of this complex and the resulting cryo-EM structure validates the previously determined crystal structures by showing that the intermolecular interactions observed between hsCLDN-4 and cCpE are conserved in both^19^. The consistency in structures determined by two independent methods therefore provides verification that the cCpE-binding and NPLVA^153^ motifs of claudins direct recognition and high-affinity binding by CpE, which we exploited to develop the sFab-driven platform. The discovery of a sFab that binds to the extracellular surfaces of claudins with subtype-preference could provide new strategies to modulate their barrier-forming properties via disruption of the claudin/claudin cis and trans interactions used to fortify tight junctions^1^. These insights and further improvements can be applied to advance the development of therapeutics to tune permeability of tissue barriers or to visualize and destroy claudin over-expressing cancers, two current applications.

The cryo-EM structures reveal the basis of COP-1 binding to claudins. They show that COP-1s CDR-H3 uses a string of four sequential Trp residues that π-π stack to form an amphipathic helix that abuts and penetrates detergent belts, giving COP-1 access to bind the hydrophobic TM regions of claudins. Other CDRs on the L and H chain contribute to COP-1 recognition and binding to claudin-4, which do so without affecting claudin-4s native interactions with cCpE. Because the interactions between hsCLDN-4 and cCpE are unaltered upon COP-1 binding, COP-1 appears to conformationally react to the pre-existing hsCLDN-4/cCpE complex rather than proactively altering it to bind its epitopes. In this way, COP-1 is poised to selectively bind the conformation that claudins naturally exist in when bound to cCpE. This is validated by our results showing that the hsCLDN-4/cCpE portion of the cryo-EM structure overlays very well onto the crystal structure and that COP-1 binds better to claudin/cCpE complexes versus claudins alone (Supplementary Figs. 4, 6).

Further evidence that COP-1 is conformationally reactive to claudin/cCpE complexes is provided by COP-1 binding curves, which fit a model for heterogeneous ligands. We surmise that this is true because either COP-1 requires time to conformationally alter the structure of its CDR-H3 to sense and penetrate the detergent to bind claudins, or BLI can distinguish the individual binding events of COP-1s H and L chains, which occur at different rates. We found that this 2:1 binding mode exists for COP-1 binding to claudins alone and to claudin/cCpE complexes, which is evidence that bound cCpE does not largely affect the binding mechanism of COP-1 to claudins. In addition to CDR-H3 of COP-1, COP-1 binds hsCLDN-4’s ECS1 loops. Thus, movements outside of CDR-H3 could also account for this binding behavior. Further characterization can uncouple which explanation is experimentally valid. Nevertheless, this finding shows that the process of COP-1 binding is complex and may rely on conformational changes within COP-1 but not its targeted claudin/cCpE epitopes. Other findings reported here provide evidence that COP-1 is subtype-selective, in addition to conformationally reactive.

Our biophysical results show that COP-1 binds claudins with subtype-preferences and reveals the mechanism of this selectivity. In the absence of cCpE, COP-1 binds preferentially to hsCLDN-4, with mmCLDN-4, hsCLDN-3, mmCLDN-3, and hsCLDN-9 binding sequentially with lower affinities. This verifies that COP-1 is selective toward claudin-4 orthologs, which are 83.7% identical, over the other three subtypes, which exhibit <70% sequence identities compared to hsCLDN-4 (Supplementary Fig. 7). At 65.7%, hsCLDN-9 has the lowest sequence identity, which explains its lowest binding capacity to COP-1 of all subtypes tested. The BLI data also shows that the k_off_ rates of COP-1 binding to claudins are faster than when cCpE is bound (Supplementary Fig. 6h–i). This shows that COP-1 epitopes on claudins are likely dynamic or less accessible in the absence of cCpE. Binding of cCpE to claudins thus limits these dynamics, stabilizing COP-1 binding epitopes resulting in longer complex association times compared to claudins alone. In this way, cCpE boosts COP-1s efficacy to bind claudins in an indirect way as no direct interactions between cCpE and COP-1 are observed. These results show that COP-1 may hold promise as a claudin-4 binding and tight junction modulating molecule as it does not rely completely on cCpE to recognize and bind claudin-4. This may be an advantage or disadvantage for potential future applications—advantageous in that COP-1s lower affinity in absence of cCpE means it could bind reversibly or transiently—disadvantageous in that COP-1 may not bind tightly if claudin/claudin interactions outcompete it or shield the COP-1 binding epitopes. Yet, if COP-1 can bind single claudins outside of tight junctions, then, like cCpE, they could still alter tight junction permeability indirectly through a sequestering mechanism that prevents their reintegration into tight junctions^20,50^.

We found it interesting that COP-1 bound hsCLDN-3 with lower affinity than hsCLDN-4 because these claudins are often expressed in the same tissues and are evolutionarily the most homologous of the 27 human subtypes^51,52^. Yet, our results show that COP-1 can bind and distinguish between hsCLDN-3 and -4, which demonstrates that COP-1 may have added value as a molecular tool to probe or modify claudin structure/function under normal or CpE-induced pathogenic conditions because it can distinguish very similar claudin subtypes in vitro.

Is COP-1 selectivity governed by recognizing unique conformations of claudins or by sequence divergence in key COP-1 binding epitopes of claudins? Our findings suggest the latter. Whether unbound or bound to cCpE, hsCLDN-9 exhibits the lowest COP-1 binding despite having high affinity for cCpE—yet it also has the lowest sequence identity to hsCLDN-4 of the subtypes assayed. This suggests that COP-1s poorer binding to it reflects a lower selectivity due to sequence changes. Existing structures of hsCLDN-4, hsCLDN-9, and mmCLDN-3 bound to cCpE reveal generally subtle alterations to claudins while major structural elements remain unchanged (Supplementary Fig. 10)^19,34,38^. Of these, mmCLDN-3 exhibits the largest structural variance. Yet despite mmCLDN-3 having potentially more conformational flexibility when bound to cCpE, our biophysical evidence shows that it is hsCLDN-9 and not mmCLDN-3 that COP-1 binds poorly to. This is evidence that sequence divergence in claudins drives COP-1s selective binding to hsCLDN-4 and not binding conformations imposed by cCpE. In light of these structural and biophysical results with COP-1, this data supports a theory where cCpE binding does not significantly alter the structure of claudins but rather traps it in a specific conformation. Any conformational changes that may be induced by cCpE binding are likely minute and localized and thus well-tolerated by COP-1. This idea is validated by COP-1s ability to bind claudins bound or unbound to cCpE. Taken together, sFabs like COP-1 could be used as probes of claudin structure in vivo as detection of COP-1 binding to claudins in cells would verify that their in vivo structures approximate those visualized in vitro here via cryo-EM.

If COP-1 selectivity is driven by sequence divergence in claudins, what residues direct COP-1 recognition? The sequence identity between hsCLDN-4 to other subtypes used in this study range from 65.7% for hsCLDN-9 to 83.7% for mmCLDN-4. We show that COP-1 employs three claudin epitopes to recognize and bind. Of these, only epitopes 1 and 2 sequentially diverge between subtypes, which means they impart subtype-selective binding to COP-1 (Fig. 5c). In the seven side chains of epitope 1 the sequence identity between hsCLDN-4 and hsCLDN-3, mmCLDN-3 and mmCLDN-4 is 85.7%—whereas between hsCLDN-4 and hsCLDN-9 it is 57.1%. In the six side chains in epitope 2, the sequence identity hierarchy between hsCLDN-4 and other claudins is hsCLDN-3 (83.3%) > mmCLDN-4 (66.7%) > mmCLDN-3 (50.0%) > hsCLDN-9 (33.3%)—again hsCLDN-9 has the lowest homology. Epitope 1 contains seven consecutive amino acids that constitute the top of TM1 (Supplementary Fig. 5). The first five residues reside in the membrane and last two are extracellular. The most variable side chains in epitope 1 between subtypes are Leu23, Cys24, and Met29 (Fig. 5c). We hypothesize that the substitutions of Val23 and Leu29 in hsCLDN-9 cause low COP-1 affinity because they face CDR-H3 and, due to the shortened lengths of these hydrophobic side chains compared to Leu23/Met29 from hsCLDN-4, disrupt interactions with Trp131 and Trp132 of COP-1. Met29 of hsCLDN-4 forms a triad with Trp47 (epitope 2) and Met62 (epitope 3) and interacts with both Trp132 of COP-1 and the headgroup of LMNG. It is thus a lynchpin for hsCLDN-4/COP-1 interactions, which explains why the mutant hsCLDN-9^L29M^ exhibited near wild type hsCLDN-4 binding affinities and why the mutant hsCLDN-4^W47A^ had markedly decreased affinity for COP-1 (Fig. 5d). Our results also show that swapping the epitope 2 sequence of hsCLDN-9 for hsCLDN-4s resulted in no improvement to COP-1 binding whereas mutating hsCLDN-4s epitope 2 to poly-alanine resulted in a large loss of COP-1 binding. The former result proves that epitope 1 is the major driver of COP-1 binding because for the hsCLDN-9^VAQVV-TSQTI46^ mutant both epitopes 2 and 3 are identical to wild type hsCLDN-4, yet COP-1 binding is unchanged compared to wild type hsCLDN-9. The latter result can be explained by the hsCLDN-4^TSQT-AAAA45^ mutant having decreased binding capacity of cCpE, thus epitope 2 cannot be stabilized by cCpE binding, resulting in lower COP-1 association and faster dissociation. These results reveal that epitopes 1 and 2 coordinate to form and maintain COP-1 interactions but that epitope 1 is the primary determinant. Because no other subtype tested shares 100% sequence identity with hsCLDN-4 in epitope 1, all claudins bind COP-1 with lower affinity due to substitutions of their corresponding Leu23 or Met29 (Table 1 and Fig. 5c). These two side chains of epitope 1 are major drivers of COP-1-selective binding to claudins.

Sequence alignment of 23 human claudins reveals that the epitope 1 LCCALPM^29^ motif is highly specific to only hsCLDN-4, with no other subtype having this sequence^38^. In fact, only hsCLDN-3, -5, -6, and -9 share identity in epitope 1 above 57%. We have shown here that COP-1 can distinguish hsCLDN-4 from -3 and -9 and thus could likely do so for hsCLDN-5 and -6. Based on epitope 1 homology we hypothesize that COP-1 will bind human claudins with the following selectivity (from best to least): hsCLDN-4, -3, -5 and -6, and finally, -9. Although this prediction requires experimental validation, if valid, would prove an exciting prospect. Human claudin-3, -4, -6, and -9 all bind CpE and are involved in a variety of pathologies, while hsCLDN-5 is the gatekeeper of blood-brain barrier tight junctions^53–55^. If COP-1 can bind effectively to these claudins in vitro it could be used in vivo to probe or modulate the various physiological processes that these claudins regulate. To our knowledge, other than the evolutionary selected CpE/cCpE, COP-1 would be one of the first experimentally validated nanomolar affinity claudin binders described in the literature. Further development of sFabs based on the COP-1 scaffold could be engineered to bind each claudin more selectively, providing additional utility. As we show here that COP-1 binds hsCLDN-4 in lipid nanodiscs, which approximate the membrane bilayer found in cells with greater accuracy than detergents, our results provide proof-of-principle that COP-1 or similarly functioning sFabs could selectively target claudins in cells and thus have potential to alter the permeabilities of tight junctions in subtype- and/or tissue-specific ways like non-Fab-based molecules^56^.

In summary, we have developed, characterized the binding of, and determined a structure of a sFab called COP-1 and confirmed its selective binding to human claudin-4 over other homologous human and mouse subtypes. The structure and corresponding analyses provide the structural and mechanistic bases of COP-1s subtype-selective recognition and binding of human claudin-4, and preliminary proof-of-principle that it or similarly engineered molecules could be used to target specific claudins at their extracellular surfaces and consequently tune the molecular permeability of tight junctions in controllable ways. To date, very few synthetic or non-CpE-based molecules have been developed and experimentally validated to bind directly to claudins, and none have had their structures determined in complex with a claudin. This work therefore enhances insights and strategies for effective molecular targeting of claudins inside and outside the membrane. Moreover, it highlights the advantages of using sFabs for these purposes as they are easy to produce, monovalent, stable, inherently bind with high affinity and selectivity, and can be rationally optimized to improve these properties. These traits that sFabs exhibit provide the added benefit of enabling cryo-EM structure determination of small membrane proteins like claudins by eliminating the bottleneck of crystallization^57–59^. Yet, as we demonstrate, can resolve structures of small proteins with high resolution and precision. In full, the molecules reported here have potential to serve both experimentalists and clinicians alike that aim to elucidate or modulate the form and function of tight junctions driven by claudins.

## Materials and methods

### Expression, purification, and labeling of claudins and enterotoxins

All claudins (hsCLDN-3, -4, -9, mmCLDN-3, and -4) and enterotoxins (cCpE and CpE) were expressed and purified as described previously^19,38,39^. Briefly, all proteins contained a C-terminal decahistidine tag preceded by a thrombin cleavage site (claudin-_His10_, cCpE-_His10_, and CpE-_His10_) and were expressed in insect cells. After cell lysis, membranes were prepared for claudins by ultracentrifugation while the supernatant was used for purification of enterotoxins. Claudins were solubilized from membranes using n-dodecyl-β-D-maltopyranoside (DDM, Anatrace) and cholesteryl hemisuccinate Tris salt (CHS, Anatrace) to a final concentration of 1/0.1% (w/v). After ultracentrifugation at 100,000 xg, the supernatants were used for purification by adding NiNTA resin (ThermoFisher) to them. For most applications, the protein-bound resin was washed and proteins were released and collected from the resin by digestion with thrombin. For enterotoxins, no detergents were used while for claudins, the concentrations of DDM/CHS in the washes were 0.087/0.0087%, and 0.04/0.004% for protein release. All proteins isolated using these methods were pure as assessed by SDS-PAGE and analytical size-exclusion chromatography (SEC) using a Superdex 200 increase column equilibrated in SEC Buffer (20 mM Hepes pH 7.4, 100 mM NaCl, 1% glycerol). For claudins, SEC buffer also contained 0.04% DDM, with no CHS. These tagless post-NiNTA proteins were frozen in liquid nitrogen at 1 mg/mL and stored at -80°C until use.

For structural studies, hsCLDN-4 was solubilized using 1/0.1% DDM/CHS, ultracentrifuged, and bound to NiNTA resin as above. However, during NiNTA purification, it was exchanged from DDM/CHS to 2,2-didecylpropane-1,3-bis-β-D-maltopyranoside (LMNG, Anatrace)/CHS by collecting the resin with a bead-capture column (Bio-Rad) then washing it for 10 column volumes each of Wash Buffer A (50 mM Tris pH 7.4, 500 mM NaCl, 20 mM imidazole, 10% glycerol, and 0.1/0.01% LMNG/CHS) then Wash Buffer B (50 mM Tris pH 7.4, 300 mM NaCl, 40 mM imidazole, 5% glycerol, and 0.1/0.01% LMNG/CHS). Beads were captured and washed for two column volumes with Cleavage Buffer (50 mM Tris pH 8.0, 150 mM NaCl, and 0.05/0.005% LMNG/CHS). After washing, 3 mL of Cleavage Buffer were added to resin and hsCLDN-4 was released by thrombin overnight. The next day, hsCLDN-4 in LMNG/CHS were captured in the flow-through, analyzed by SDS-PAGE and analytical SEC, and used to form complexes for cryo-EM analysis.

For biophysical studies, claudins and/or enterotoxins required immobilization on biosensors. For claudins, the tagless post-NiNTA pure samples were biotinylated while enterotoxins were eluted off NiNTA using imidazole to maintain their C-terminal decahistidine tags. Briefly, claudins were purified as above and equilibrated in Biotin buffer (20 mM Hepes pH 7.2, 150 mM NaCl, 1% glycerol, and 0.04% DDM). This pH specifically labels the N-terminal amine of claudins using NHS chemistry. Claudins were labeled with NHS-PEG4-Biotin (ThermoFisher) using 4:1 biotin:claudin molar excess overnight at 4°C. The next day, 50 mM Tris pH 7.4 was added to quench the reaction and the samples were buffer exchanged into BLI buffer (20 mM Tris pH 7.4, 100 mM NaCl, 1% glycerol, and 0.03% DDM) using PD-10 columns (Bio-Rad). For enterotoxins, the proteins were released from NiNTA resin after incubation with Elution buffer (50 mM Tris pH 7.4, 150 mM NaCl, 300 mM imidazole, and 1% glycerol). Enterotoxins were then buffer exchanged into BLI buffer using a PD-10 column to remove imidazole. All claudin-_Biotin_ and cCpE-_His10_ and CpE-_His10_ proteins were analyzed by SDS-PAGE and analytical SEC and judged to be biochemically homogenous and pure; at which point they were frozen in liquid nitrogen at 1 mg/mL and stored at -80°C until use.

### Development, validation, expression, and purification of COP-1

COP-1 was developed using a phage display library encoding synthetic antigen-binding fragments (sFabs) concurrent with previously reported sFabs COP-2 and COP-3^39,60^. Preparation of hsCLDN-4/cCpE complexes are provided therein. For panning, DDM-solubilized and biotinylated hsCLDN-4 in complex with cCpE was used as input into the phage display pipeline after polishing and removing free biotin using SEC. COP-1 was therefore developed, validated, expressed, and purified identically to previously described methods^39^. During purification, COP-1 was found to be less soluble in buffers than COP-2 and -3, and so 0.01% DDM was added during affinity purification. After affinity purification, COP-1 was exchanged to BLI buffer, frozen in liquid nitrogen, and stored at -80°C until use in biochemical, biophysical, and structural biology studies.

### Preparation of complexes for cryo-EM

HsCLDN-4/cCpE complexes were formed using hsCLDN-4 in LMNG/CHS (250 µg) and 1.2 molar excess of tagless cCpE incubated at 4°C for 1 h. COP-1 was then added at a 1.0 molar ratio to hsCLDN-4 and incubated with the hsCLDN-4/cCpE complex at 4°C overnight. The next day, 1.5 molar excess of V_H_H nanobody (Nb) to COP-1 was added and incubated at 4 °C overnight. The assembled hsCLDN-4/cCpE/COP-1/Nb complexes were then concentrated using a 100 kDa MWCO concentrator (Millipore), 0.2 µm filtered, then loaded onto a Superdex 200 increase 10/300 column equilibrated in Cryo buffer (20 mM Hepes pH 7.4, 100 mM NaCl, and 0.003% LMNG). Fractions from SEC were run on SDS-PAGE and those containing the hsCLDN-4/cCpE/COP-1/Nb complex was pooled and concentrated to 7 mg/mL using a 100 kDa MWCO concentrator. A fraction of this sample was also diluted in half to 3.5 mg/mL using Cryo buffer so that two concentrations could be tested in vitrification. Samples were shipped to the University of Chicago overnight on ice for vitrification and cryo-EM analysis.

Samples of a hsCLDN-4/cCpE/COP-1 complex lacking the Nb were also prepared as described above. This complex was pooled and concentrated to 6 mg/mL then shipped overnight on ice to the Pacific Northwest Cryo-EM Center (PNCC) for vitrification and cryo-EM analysis.

### Cryo-EM vitrification, data collection, and processing

Once received, samples of the hsCLDN-4/cCpE/COP-1/Nb complex were placed on ice and vitrified the same day. Grids for cryo-EM analyses were glow-discharged for 30 s at 20 W using a Solarus 950 (Gatan) plasma cleaner then vitrified using a Vitrobot Mark IV (ThermoFisher) plunge freezing apparatus. Aliquots (3.5 μl) of hsCLDN-4/cCpE/COP-1/Nb at both 7 and 3.5 mg/mL were each applied to a Quantifoil R1.2/1.3 200 mesh grid, an Au-Flat 0.6/1.0 300 mesh grid, and a UltrAuFoil 0.6/1.0 300 mesh grid at 8 °C and 100% relative humidity. Grids were blotted for 5 s with a blot force of 2 and plunge frozen into liquid ethane cooled by liquid nitrogen. Grids were stored in liquid nitrogen before imaging. The six grids were screened for thin ice and particle presence and distribution. The 7 mg/mL sample frozen on UltrAuFoil 0.6/1.0 300 mesh grid was used for data collection.

Cryo-EM data collection for the hsCLDN-4/cCpE/COP-1/Nb complex was performed on a Titan Krios G3i (ThermoFisher) equipped with a Gatan K3 direct electron detector and BioQuantum GIF at the University of Chicago Advanced Electron Microscopy Core Facility (RRID:SCR_019198). 5039 movies were collected using EPU (ThermoFisher) in CDS mode at 81,000× magnification with a super resolution pixel size of 0.534 Å and physical pixel size of 1.068 Å, defocus range of 0.9 to 2.1 μm using a step of 0.2 μm, with a total dose of 60 electron/Å^2^ fractionated over 50 total frames.

Once the hsCLDN-4/cCpE/COP-1 complex was received, it was also placed on ice then vitrified the same day. Grids for cryo-EM analyses were glow-discharged for 60 s at 15 mA in a Pelco easiGlow (Ted Pella Inc) instrument then vitrified using a Vitrobot Mark IV (ThermoFisher) plunge freezing apparatus. Aliquots (3 μl) of hsCLDN-4/cCpE/COP-1 were applied to a Quantifoil R2/1 200 mesh grid at 4 °C and 100% relative humidity. The grid was blotted for 1.5 s with a blot force of 2 and plunge frozen into liquid ethane cooled by liquid nitrogen, then stored in liquid nitrogen prior to screening and data collection.

Cryo-EM data collection for the hsCLDN-4/cCpE/COP-1 complex was performed on a Titan Krios G3i (ThermoFisher) equipped with a Gatan K3 direct electron detector and BioContinuum GIF at PNCC. 1073 movies were collected using SerialEM in counting mode at 92,000× magnification with a super resolution pixel size of 0.2535 Å and physical pixel size of 0.5070 Å, defocus range of 0.8 to 2.2 μm using a step of 0.2 μm, with and a total dose of 60 electron/Å^2^ fractionated over 50 total frames.

All micrograph and particle processing for both complexes were performed in CryoSPARC^61^. Patch-motion correction and patch-CTF correction were used to correct for beam-induced motion and calculate CTF parameters from the motion-corrected micrographs. Blob-based template picking followed by 2D classification were used to generate templates that were subsequently used for template-based particle picking. Particles identified from this template-based picking procedure were subjected to at least one round of 2D classification, followed by ab initio 3D reconstruction, heterogeneous and homogenous refinement, and finally non-uniform refinement. Local resolution was estimated by CryoSPARC’s algorithms using half maps. For the hsCLDN-4/cCpE/COP-1/Nb complex, the reported 2.62 Å map is a result of CryoSPARC implemented masking that included the entire complex and detergent micelle, which we term as a “loose mask” map (EMD-41899). This map was used for all subsequent model building and refinement of the complex, which was ultimately uploaded to the PDB. Using Chimera, we manually set a map threshold that minimized the detergent micelle to create a “tight mask” that encapsulated the entire protein complex without detergent micelle^62^. This mask was used for non-uniform refinement in CryoSPARC to generate the 2.17 Å map (EMD-41897). Although the resolution of this map was higher, we did not use it for model building or interpretation, rather to illustrate that cryo-EM-generated data has higher resolution potential for claudins. For the hsCLDN-4/cCpE/COP-1 complex, default masking in Cryosparc was used to generate the 4.2 Å maps (EMD-41915). A summarized workflow is shown in Supplementary Fig. 2 for all three maps.

### Model building, refinement and structure determination

A 3D model of COP-1 was generated using AlphaFold2^63^. Then, the crystal structure of hsCLDN-4 bound to cCpE (PDB ID 7KP4) and the COP-1 model were independently placed into the cryo-EM density manually then fit into the map with Chimera. The sFab/Nb complex from PDB ID 7ZLG that contains a sFab L chain-binding V_H_H nanobody^41^ was then superposed onto COP-1 by aligning the L chains of the sFab and COP-1. The 3D coordinates of the Nb and COP-1 were then written into a .pdb file containing this orientation, as well as the fit model of hsCLDN-4/cCpE from the crystal structure, creating a file with all four proteins and their respective five chains. Each chain was then rigid body and real space refined into the 2.62 Å “loose” masked cryo-EM map using Coot^64^. Refinement of the model was done iteratively using Namdinator and phenix.real_space_refine to optimize model-to-map fit to the “loose” masked map, resulting in the final structural model of the hsCLDN-4/cCpE/COP-1/Nb complex (PDB ID 8U4V)^47,65^. This model was refined against both the “loose” and “tight” maps to generate the data in Table 2, but only the model refined against the “loose” masked map was deposited to the PDB as no significant changes resulted using the “tight” masked map. The hsCLDN-4/cCpE/COP-1 complex structure was built using the claudin-4/cCpE/COP-1/Nb complex model after deleting the Nb and real-space refined against its map to generate the final model (PDB ID 8U5B). The programs used to visualize and build the structures included Coot and Chimera, refinement in Phenix, and figures were made using Chimera—using the SBGrid Consortium Software Suite^66^. Table 2 and Supplementary Fig. 11 shows data collection, refinement, and validation statistics for the two structural models.

**Table 2:** Cryo-EM Data Collection, Refinement and Validation Statistics

	hsCLDN-4/cCpE/COP-1/Nb PDB ID: 8U4V EMDB-41899 (loose) and EMDB-41897 (tight)		hsCLDN-4/cCpE/COP-1 PDB ID: 8U5B EMDB-41915
**Collection & Processing**			
Magnification	81,000		92,000
Voltage (kV)	300		300
Electron exposure (e–/Å^2^)	60		60
Defocus range (μm)	0.9–2.1		0.8–2.2
Pixel size (Å)	1.068		0.507
Symmetry imposed	C1		C1
Number of micrographs	5,039		1,071
Initial particle images (no.)	7,702,466		565,063
Final particle images (no.)	1,126,378		37,296
Map resolution (Å)	2.62	2.17	4.20
FSC threshold	0.143	0.143	0.143
Mask	Loose	Tight	
Map resolution range (Å)	12.5–2.4	9.7–2.1	33.2–3.7
**Refinement**			
Initial model used (PDB ID)	7KP4	7KP4	8U4V
Model resolution (Å)^a^	2.80/2.90	2.40/2.40	4.50 / 4.70
FSC threshold	0.50	0.50	0.50
Sharpening *B-*factor (Å^2^)	−105	−54	−155
Dimensions (Å)	79.96, 95.05, 128.16	79.03, 95.05, 126.02	64.39, 93.80, 129.79
Chains	7	7	5
Atoms	6637	6637	5721
Residues	862	862	745
*B-*factors (Å^2^)			
Protein	117.45	69.27	238.34
Ligand	150.33	114.19	270.75
Water	99.67	70.10	*N/A*
R.M.S. deviations			
Bond lengths (Å)	0.007	0.007	0.008
Bond angles (°)	1.142	1.193	1.144
**Validation**			
MolProbity score	1.81	1.90	1.84
Clashscore	8.66	10.78	11.58
Poor Rotamers (%)	1.80	1.52	0.00
Ramachandran plot			
Favored (%)	97.18	96.71	96.20
Allowed (%)	2.82	3.29	3.80
Disallowed (%)	0.00	0.00	0.00

*N/A* not applicable.

^a^d Fourier Shell Coefficient (FSC) Model 0.50 Masked/Unmasked from Phenix.

### Biophysical characterization

For biophysical analyses, we used claudin-_Biotin_, tagless cCpE, cCpE-_His10_, and CpE-_His10_ and post-affinity-purified COP-1 all solubilized in BLI buffer (20 mM Tris pH 7.4, 100 mM NaCl, 1% glycerol, and 0.03% DDM). Bio-layer interferometry (BLI) was performed at 25°C in 96-well black flat bottom plates (Greiner) using an acquisition rate of 5 Hz averaged by 20 using an Octet© R8 BLI System (FortéBio/Sartorius), with assays designed and setup using Blitz Pro 1.3 Software. Binding experiments consisted of the following steps: sensor equilibration (30 s), loading (120 s), baseline (60 s), and association and dissociation (200–300 s each).

The first experiments consisted of validating claudin binding to cCpE. Here, 500 nM of cCpE-_His10_ was immobilized on NiNTA (Dip and Read) sensors and associated with 0–600 nM claudin-_Biotin_ (Supplementary Fig. 6a–e). Association and dissociation were done for 300 s to compare to previously reported results. We next tested CpE binding to claudins where 500 nM of CpE-_His10_ was immobilized on NiNTA sensors and associated with 200 nM claudin-_Biotin_ (Supplementary Fig. 6f). These results were comparable to cCpE. All subsequent associations and dissociations were done for 200 s. For cCpE binding to COP-1, 500 nM of cCpE-_His10_ was immobilized in NiNTA biosensors and binding was tested against 0–750 nM COP-1 (Supplementary Fig. 6g). For COP-1 binding to claudins, 1 µM of claudin-_Biotin_ was immobilized on Streptavidin-SA (Dip and Read) biosensors and binding was tested against 500 nM COP-1 (Supplementary Fig. 6h). General trends for COP-1 binding to claudin/enterotoxin complexes were then performed as follows. First, 300 nM cCpE-_His10_ was incubated with 300 nM of claudin-_Biotin_ at 20°C for 1 h to make complexes and then this 600 nM of pre-formed claudin-_Biotin_/cCpE-_His10_ complexes were immobilized on NiNTA biosensors and binding was tested against 250 nM COP-1 (Supplementary Fig. 6i). This same method was used to test COP-1 (250 nM) binding to 600 nM pre-formed claudin-_Biotin_/CpE-_His10_ complexes (Supplementary Fig. 6j). Once general trends were established, we obtained the full binding and kinetic constants of claudin/cCpE against COP-1 as follows: 600 nM pre-formed claudin-_Biotin_/cCpE-_His10_ complexes were immobilized on NiNTA sensors then associated with 0–750 nM COP-1 (Supplementary Fig. 6k–o). This strategy was chosen over immobilizing pre-formed claudin-_Biotin_/cCpE-_His10_ complexes on SA sensors after it was determined that NiNTA sensors resulted in greater signal strength. Here, 1200 nM of pre-formed hsCLDN-4-_Biotin_/cCpE-_His10_ complexes were immobilized on SA biosensors and binding was tested against 0-750 nM COP-1, which resulted in similar dissociation constants compared to those obtained from NiNTA sensors (Supplementary Fig. 6p). Immobilizing half the concentration of hsCLDN-4/cCpE complex resulted in ~5-fold higher binding signal. We attribute this to the relatively small NiNTA moiety compared to the larger streptavidin (52 kDa), which results in more complex bound per sensor and thus greater light interference upon COP-1 binding. Mutant claudin-_Biotin_ experiments were performed identically using the NiNTA strategy with pre-formed cCpE-_His10_ complexes. Results for multi-point quantitative analyses appear in Supplementary Fig. 6 and Supplementary Table 2 while those for single-point qualitative analyses appear in Supplementary Fig. 6 and Supplementary Table 3. Supplementary Table 4 highlights the complete BLI workflow.

For claudin binding to enterotoxin measurements the time courses for association and dissociation were fit to a 1:1 binding model using the Octet^®^ Analysis Studio (Sartorius). COP-1 binding to claudins and claudin/enterotoxin complexes measurements, however, did not fit a 1:1 binding model, so a 2:1 heterogenous ligand model was used. This model assumes COP-1 binds at two independent antigen sites with different rate constants, which can be explained by independent binding of each COP-1 chain to the complex or two binding modes that rely on a conformational switch in the claudin/cCpE complex to expose the COP-1 binding epitope. Thus, two affinity and rate constants are reported. At the protein concentrations used, no significant non-specific binding of claudins or COPs to NiNTA or SA sensors were detected.

### Biochemical characterization

For biochemical analyses we used samples prepared as described above in biophysical analyses. Here, 300 μL of 600 nM pre-formed claudin-_Biotin_/cCpE-_His10_ complexes (~7 μg) were extracted from 1.5 wells of the 96-well plate. These samples, representing five claudin/cCpE complexes, were injected onto a TSKgel QC-PAK GFC 300 SEC column (Tosoh Bioscience) equilibrated in BLI buffer and run for 18 min at a flow rate of 0.5 mL/min. Another 300 μL of these same five claudin/cCpE complexes were added to a microcentrifuge tube and 1 molar excess COP1 (~10 μg) was added. These samples were nutated at 20 °C for 1 h as with BLI to form complexes. After time, these samples, representing five claudin/cCpE/COP-1 complexes, were injected onto the SEC column. As a control, 300 μL of 600 nM pre-formed hsCLDN-4-_Biotin_/cCpE-_His10_ complexes was bound to 1 molar excess of COP-2, a cCpE-binding sFab, incubated at 20 °C for 1 h, then injected on SEC. Retention of complexes was assessed by observing decreases to the elution times of claudin/cCpE/sFab complexes compared to uncomplexed claudin/cCpE peak fractions.

### Claudin mutant and mimetic binding analyses

The pFastBac1 cloned plasmids containing human claudin-4-_His10_ and human claudin-9-_His10_ were altered using site-directed mutagenesis. Mutants were generated with the following forward primers and their equivalent reverse compliments:

- hsCLDN-4^LPM-AAA29^ 5′-atgctgtgctgcgcggcggccgcgtggcgcgtgacggc-3′
- hsCLDN-4^W47A^ 5′-cctcgcagaccatcgcggagggcctatgga-3′
- hsCLDN-4^TSQT-AAAA45^ 5′-tcggcagcaacattgtcgccgcggcggccatctgggagggccta-3′
- hsCLDN-9^L29M^ 5′-ctgcgccctgcccatgtggaaggtgac-3′
- hsCLDN-9^VAQVV-TSQTI46^ 5′-ccttcatcggcaacagcatcgtgacgagccagacgatatgggagggcctgtggatgtcctg-3′

Protocols for expression and purification of all mutants were identical to those of wild type claudin-4-_His10_ and human claudin-9-_His10_ referenced above. Post-IMAC pure and tagless mutant claudins were assessed for purity and homogeneity using SDS-PAGE and SEC, then these proteins were used for binding analyses.

In addition, wild type full-length hsCLDN-4 purified in DDM was exchanged from detergent into amphipol polymers and lipoprotein nanodiscs for binding studies. Here, amphipol A8-35 (Anatrace) was added to detergent soluble hsCLDN-4 at a 4:1 mass-to-mass ratio and incubated on ice for 30 min. After time, 300 mg of methanol washed SM-2 biobeads (Bio-Rad) were added to remove excess detergent overnight at 4 °C. For nanodisc incorporation, detergent soluble hsCLDN-4 was mixed with soy polar or eggPC lipids (Avanti), and the membrane scaffolding protein (MSP) 1D1 with its histidine tag removed at a 1:80:4 molar ratio, allowed to incubate on ice for 1 h, then 500 mg of methanol washed SM-2 biobeads were added and incubated overnight at 4 °C. Post-detergent exchanged hsCLDN-4s were assessed for purity and homogeneity using SDS-PAGE and SEC. Invariably, nanodiscs without incorporated hsCLDN-4 were present in these preparations. To estimate to amount of hsCLDN-4^Nanodisc^, we added cCpE and COP-1, ran SEC, and quantified the percent containing hsCLDN-4 by elution time changes. These proteins were isolated in BLI buffer without DDM and used for binding analyses.

The first binding analyses consisted of testing DDM-soluble mutant claudins and non-detergent hsCLDN-4 binding to cCpE. Here, 500 nM of cCpE-_His10_ was immobilized on NiNTA sensors and associated with 200 nM mutant hsCLDN-4, mutant hsCLDN-9, and non-detergent hsCLDN-4, in single-point analyses similar to what has been presented in Supplementary Fig. 6f. Association and dissociation were done for 300 s (Supplementary Fig. 9a, c, e). For hsCLDN-4^Nanodiscs^, 200 nM and 400 nM protein were tested to validate concentration-dependent binding, and dissociated was extended to 600 s. The hsCLDN-4 mutant LPM-AAA^29^ was not able to bind cCpE. Several attempts to purify and stabilize this mutant were unsuccessful despite protein bands being visualizing at the appropriate molecular weight via SDS-PAGE and correct elution times on SEC. This mutant does not express as well as others and it was concluded must possess an altered structure of its ECS and thus was not tested further. The next single-point binding studies tested the binding of COP-1 to pre-formed mutant hsCLDN-4, mutant hsCLDN-9, and non-detergent hsCLDN-4 complexes with c/cCpE-_His10_. As before, 300 nM cCpE-_His10_ was incubated with 300 nM mutant hsCLDN-4, mutant hsCLDN-9, or non-detergent hsCLDN-4 at 20 °C for 1 h, and then this 600 nM of pre-formed claudin/cCpE-_His10_ complexes were immobilized on NiNTA biosensors and binding was tested against 400 nM COP-1 similar to what has been presented in Supplementary Fig. 6i. As we expected mutants to have altered binding capacity for COP-1, we used 400 nM versus 200 nM as done previously. For hsCLDN-4^Nanodiscs^, 150 nM and 300 nM protein were co-complexed with 300 nM cCpE-_His10_ to validate concentration-dependent binding. Association and dissociation were done for 200 s (Supplementary Fig. 9b, d, f). For claudin mutants, all binding was done in buffers consisting of BLI buffer with 0.03% DDM, while for non-detergent hsCLDN-4, all binding was tested in BLI buffer without DDM. hsCLDN-4^Amphipol^ and hsCLDN-4^Nanodisc^ binding to unloaded NiNTA sensors showed no non-specific binding, and COP-1 also exhibited no significant binding signal to unloaded NiNTA sensors or cCpE-loaded sensors with no claudin in absence of detergent at 400 nM. Data for claudin mutant and non-detergent claudin binding to cCpE were fit to a 1:1 binding model while binding of these proteins to COP-1 were fit to a 2:1 binding model using Octet^®^ Analysis Studio (Sartorius). Thus, one and two affinities and rate constants are reported, respectively. Data appears in Supplementary Fig. 9 and Supplementary Table 3.

### Statistics and reproducibility

Table legends and experimental details in the *Materials and Methods* describe the number of replicates and statistical analyses performed in the BLI analyses. Reproducibility of these BLI analyses is demonstrated by the strong agreement in results obtained from single versus multi-point, analysis, with the latter being more statistically robust.

### Reporting summary

Further information on research design is available in the Nature Portfolio Reporting Summary linked to this article.

### Supplementary information

Supplementary Information

Reporting Summary

## Data Availability

The cryo-EM structure of hsCLDN-4/cCpE/COP-1/Nb has accession code 8U4V in the Protein Data Bank (PDB) and cryo-EM maps of this complex have been deposited to the Electron Microscopy Data Bank (EMDB) under accession codes EMD-41899 (loose mask map) and EMD-41897 (tight mask map). The cryo-EM structure of hsCLDN-4/cCpE/COP-1 has PDB accession code 8U5B and EMDB accession code EMD-41915.
