# Supplementary material for: Structural and biophysical insights into targeting of claudin-4 by a synthetic antibody fragment
Source: Commun Biol. 2024 Jun 17;7:733. doi: 10.1038/s42003-024-06437-6 (PMC11183071; doi:10.1038/s42003-024-06437-6)
Supplement: Supplementary file 1 — Supplementary Information [file 42003_2024_6437_MOESM1_ESM.pdf]

Supplementary Information for
Structural and biophysical insights into targeting of claudin-4 by a synthetic antibody fragment

Satchal K. Erramilli[1], Pawel K. Dominik[1,a], Chinemerem P. Ogbu[2,b], Anthony A. Kossiakoff[1], and Alex J. Vecchio[2,b,#,*]

[1]Department of Biochemistry and Molecular Biology, University of Chicago, Chicago, IL, 60637 USA
[2]Department of Biochemistry, University of Nebraska-Lincoln, Lincoln, NE, 68588 USA
[a]Present address: Pfizer, San Diego, CA, 92121 USA
[b]Present address: Department of Structural Biology, University at Buffalo, Buffalo, NY, 14203 USA
[#]Lead author
*Corresponding author

ORCID: 0000-0001-8694-7681 (S.K.E.); 0000-0001-5663-7148 (C.P.O); 0000-0003-3174-9359 (A.A.K); 0000-0002-4222-7874 (A.J.V)

Email: vecchioa@buffalo.edu

This PDF file includes:

Extended Discussion
Supplementary Figures S1 to S11
Supplementary Tables S1 to S4
Supplementary References

Extended Discussion

Claudin-4's "targetable" drug binding surface

The hsCLDN-4/cCpE/COP-1/Nb complex structure determined using cryo-EM sheds light on the boundary of membranes and allows visualization of detergents to resolve the solvent-accessible surface of claudins. The visualized LMNG detergent belt around hsCLDN-4 allows us to experimentally establish the regions of hsCLDN-4 that reside inside and outside membranes, which has only been able to be estimated previously from crystal structures. We show that the membrane boundary approximated by *in silico* methods using just the structural coordinates agree with the experimental evidence provided by the detergent belt visualized by cryo-EM. The detergent belt and membrane boundaries reveal that 113 amino acids or ~12,121 Da (54%) of claudin-4s tertiary structure resides outside of the membrane to provide antigenic or binding surfaces for potential drugs. When considering non-terminal structured domains in which a molecule could orient and bind, only 83 amino acids or ~9,126 Da (40%) of claudin-4 is "targetable" by an agent. This insight provides critical information for *in silico*-based structure-guided design of claudin binding molecules. COP-1s mechanism for penetrating the hydrophobic environment to access typically inaccessible regions of claudin-4 notably expands the targetable surface of claudins. COP-1 is therefore an innovative scaffold for these and other efforts to design claudin-binding molecules.

A detergent binding site on claudin-4

Cryo-EM maps of the 2.6 Å hsCLDN-4/cCpE/COP-1/Nb and 4.2 Å hsCLDN-4/cCpE/COP-1 structures both show strong density at hsCLDN-4/COP-1 CDR-H3 interfaces, which we modeled a LMNG detergent into. The branched maltoside headgroup of LMNG is sandwiched between Phe103 and Phe113 of COP-1s H chain and Met29, Trp47, and Met62 of hsCLDN-4 (**Fig. 3**). LMNG seemingly facilitates indirect interactions between hsCLDN-4 and COP-1. Although LMNG was not used for sFab panning, the structurally and chemically analogous detergent DDM was. The maltoside headgroups of DDM and LMNG are identical, and thus this interface could be conserved in the presence of either detergent or any other maltosidic or oven glucosidic detergent. The ability

to visualize and model a normally dynamic molecule like a detergent, holds promise to use cryo-EM to resolve other important tight junction modulating molecules, like cholesterol, lipids, or small molecules like drugs, bound to claudins.

Our data suggests that LMNG and/or DDM, although present at the interface, do not contribute strongly to COP-1 binding as COP-1 is able to bind with similar affinities to amphipol or nanodisc-incorporated hsCLDN-4 as in DDM solubilized. (**Supplementary Fig. 9**). However, the kinetics of COP-1 binding do change in these different mimetics, which in turn decreases the affinity of COP-1 for hsCLDN-4. Interestingly, the k_{on} appears to drive this change in affinity rather than k_{off}. In both amphipols and nanodiscs, the k_{on} rate was slower than it was in detergent (**Supplementary Figs. 9d** and **9f**). We suspect that this can be interpreted as COP-1 taking longer to conformationally adapt to hsCLDN-4 in these membrane mimetics. We surmise that COP-1s CDR-H3 is responsible for this delayed binding as these membrane mimetics are structurally and chemically very divergent from detergents, and potentially also less flexible to penetrate them and access the hydrophobic surface of hsCLDN-4. Once bound, however, COP-1 exhibits slow k_{off} rates, which further demonstrates its potential utility in a multitude of biochemical or structural applications.

In vitro **and** ***in vivo*** **applications for COP-1, -2 and -3**

The three sFabs we have developed bind with two distinct binding modes and thus have COP-specific utility. First mode of binding: COP-2 and -3 both bind to cCpE, but not to CpE or claudins, and are thus useful for determining structures of claudins that bind cCpE with high affinity, like claudin-3, -4, -6, -8, and -9. COP-2 and -3 bind to an epitope opposite of the claudin/cCpE interface, leaving the COP-1 binding site accessible. Unfortunately, because COP-2 and -3 do not bind CpE, they have no effect on CpE-based cytotoxicity in cells, so *in vivo* applications are less straightforward. Second mode of binding: COP-1 binds to claudins alone or in complex with cCpE or CpE, although it binds with higher affinity to the latter and binds best to hsCLDN-4. Because the COP-1 binding epitopes do not utilize enterotoxin directly, it can also be used to determine structures of claudins that bind cCpE and CpE with high affinity as aforementioned. Thus, COP-1 can enable structure determination of the yet determined claudin/CpE “small” complex that forms to initiate CpE’s cytotoxic β-pore. Because, like COP-2 and -3, COP-1 does not bind CpE, it likely will not obstruct CpE cytotoxic pore formation, although we are in the process of verifying this. We demonstrate here that because COP-1 binds claudins alone and penetrates the hydrophobic membrane environment to do so, it may have utility *in vivo*. We envision that COP-1 can be used to probe the structure and function of claudins it binds in cells, like hsCLDN-4, mmCLDN-3 and mmCLDN-4, or potentially disrupt lateral claudin interactions since we show here how it binds and that it exhibits preferential binding to these claudin subtypes. Through affinity and selectivity maturation, COP-1 could be engineered further to improve its *in vitro* and *in vivo* functions to create a tight junction modulating molecule.

Supplementary, Figures and Tables

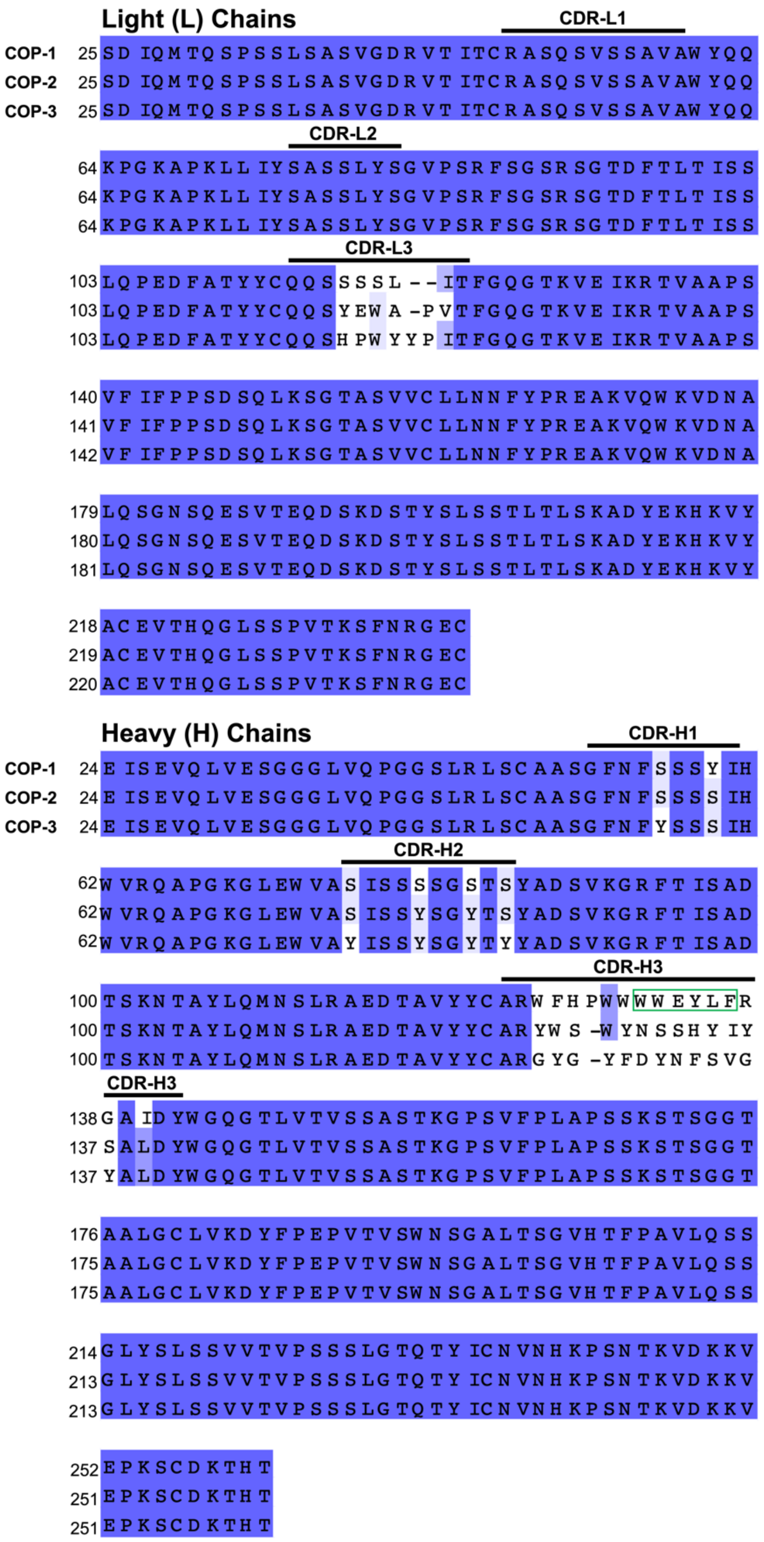

Supplementary Fig. 1. Sequence Alignment of COPs. The L and H chains of COP-1, -2, and -3 were aligned and highlighted within are the CDR-1-3 regions. The amphipathic helix sequence of CDR-H3 in COP-1 in boxed (green).

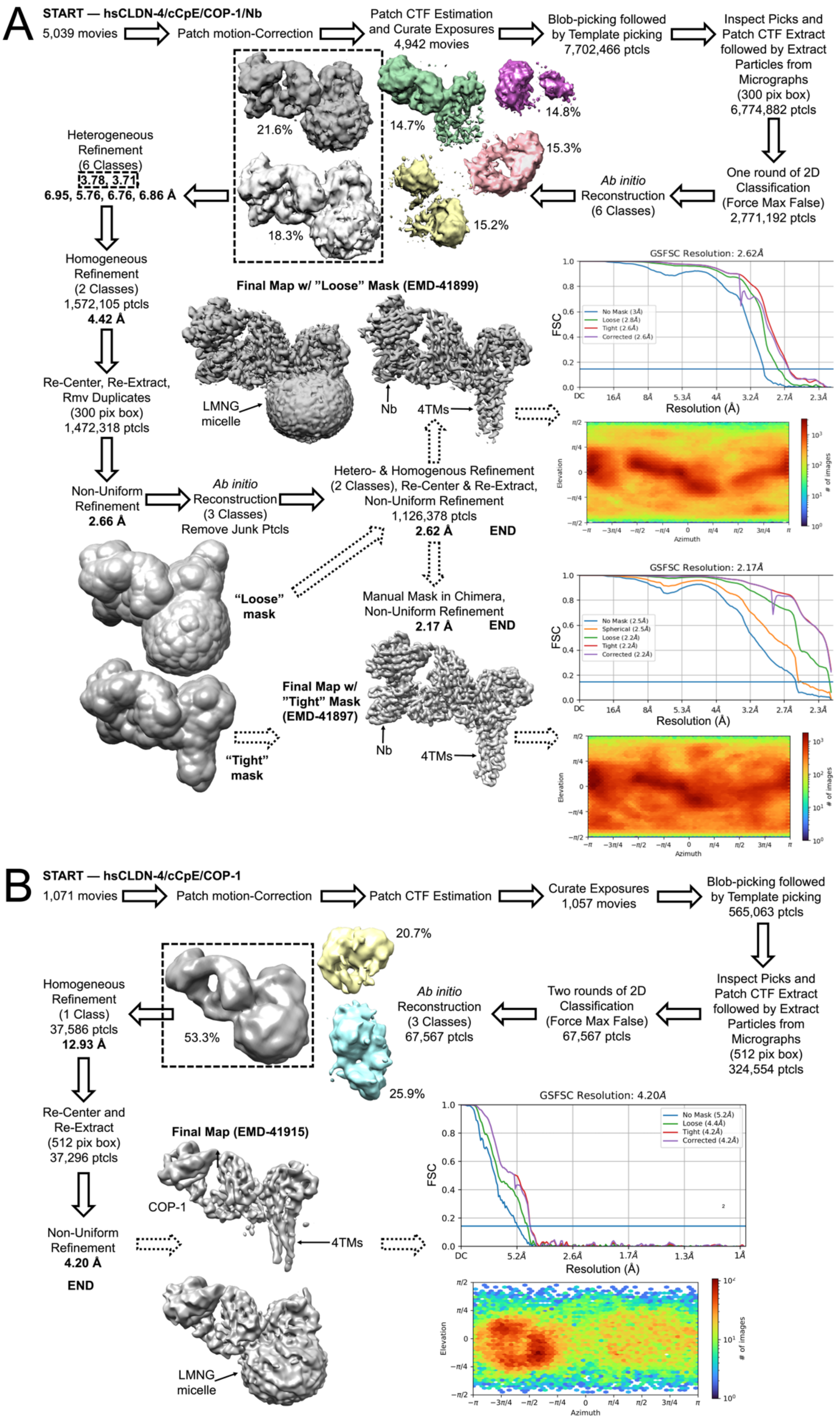
A
START — hsCLDN-4/cCpE/COP-1/Nb
5,039 movies
Patch motion-Correction
Patch CTF Estimation and Curate Exposures 4,942 movies
Blob-picking followed by Template picking 7,702,466 ptcls
Inspect Picks and Patch CTF Extract followed by Extract Particles from Micrographs (300 pix box) 6,774,882 ptcls
One round of 2D Classification (Force Max False) 2,771,192 ptcls
Ab initio Reconstruction (6 Classes)
21.6%
14.7%
14.8%
15.3%
18.3%
15.2%
Heterogeneous Refinement (6 Classes)
3.78, 3.71
6.95, 5.76, 6.76, 6.86 Å
Homogeneous Refinement (2 Classes) 1,572,105 ptcls
4.42 Å
Re-Center, Re-Extract, Rmv Duplicates (300 pix box) 1,472,318 ptcls
Non-Uniform Refinement
2.66 Å
Ab initio Reconstruction (3 Classes) Remove Junk Ptcls
Hetero- & Homogenous Refinement (2 Classes), Re-Center & Re-Extract, Non-Uniform Refinement 1,126,378 ptcls
2.62 Å
END
Final Map w/ "Loose" Mask (EMD-41899)
LMNG micelle
Nb
4TMs
GSFSC Resolution: 2.62Å
No Mask (3Å)
Loose (2.8Å)
Tight (2.6Å)
Corrected (2.6Å)
FSC
Resolution (Å)
Elevation
Azimuth
# of images
"Loose" mask
Manual Mask in Chimera, Non-Uniform Refinement
2.17 Å
END
GSFSC Resolution: 2.17Å
No Mask (2.5Å)
Spherical (2.5Å)
Loose (2.2Å)
Tight (2.2Å)
Corrected (2.2Å)
FSC
Resolution (Å)
Final Map w/ "Tight" Mask (EMD-41897)
Nb
4TMs
"Tight" mask
Elevation
Azimuth
# of images
B
START — hsCLDN-4/cCpE/COP-1
1,071 movies
Patch motion-Correction
Patch CTF Estimation
Curate Exposures 1,057 movies
Blob-picking followed by Template picking 565,063 ptcls
Inspect Picks and Patch CTF Extract followed by Extract Particles from Micrographs (512 pix box) 324,554 ptcls
Two rounds of 2D Classification (Force Max False) 67,567 ptcls
Ab initio Reconstruction (3 Classes) 67,567 ptcls
20.7%
53.3%
25.9%
Homogeneous Refinement (1 Class) 37,586 ptcls
12.93 Å
Re-Center and Re-Extract (512 pix box) 37,296 ptcls
Non-Uniform Refinement
4.20 Å
END
Final Map (EMD-41915)
COP-1
4TMs
LMNG micelle
GSFSC Resolution: 4.20Å
No Mask (5.2Å)
Loose (4.4Å)
Tight (4.2Å)
Corrected (4.2Å)
FSC
Resolution (Å)
Elevation
Azimuth
# of images

Supplementary Fig. 2. Cryo-EM Data Processing Workflows for hsCLDN-4/cCpE/COP-1 Complexes. (a) Workflow for the hsCLDN-4/cCpE/COP-1/Nb complex. Note the same particles were subjected to the same final non-uniform refinement but the input masks varied, resulting in different final resolution maps. (b) Workflow for the hsCLDN-4/cCpE/COP-1 complex. Fourier Shell Correlation (FSC) curves from gold-standard refinement in CryoSPARC of both complexes are shown with the 0.143 FSC cutoff indicated by line (blue). Plots of the angular distribution of particles in the final refinement are shown below FSC curves.

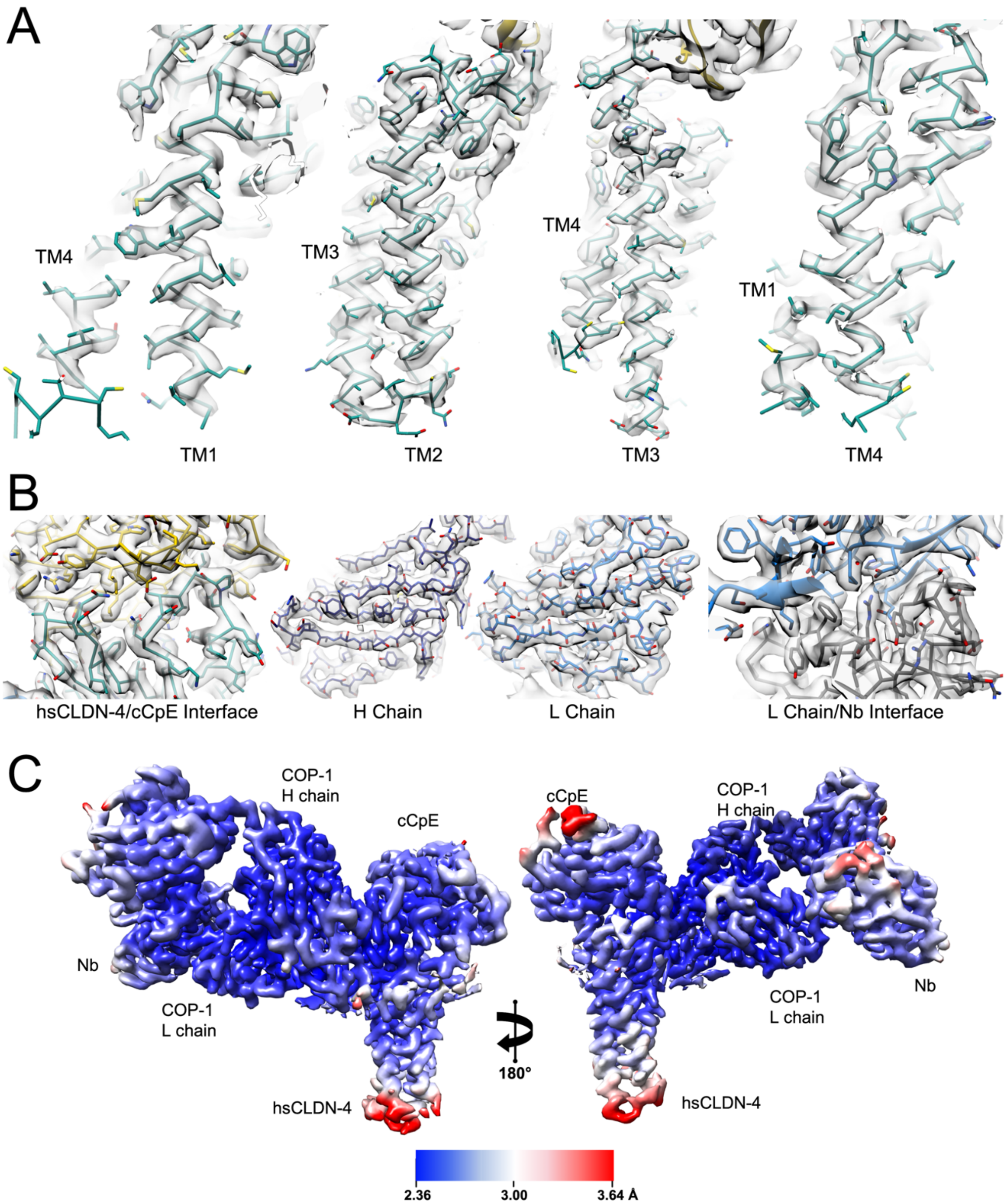

Supplementary Fig. 3. High Resolution Features of the hsCLDN-4/cCpE/COP-1/Nb 2.6 Å Map. (a) Final “loose” masked cryo-EM map (light grey) of the hsCLDN-4 TM region (teal) within the LMNG belt. Depicted are the map features around the four α-helical TMs. (b) Final “loose” masked cryo-EM map of extracellular areas. Depicted from left-to-right are the map features (light grey) around the hsCLDN-4 (teal) and cCpE (yellow) interface; COP-1 H chain β-sheets (dark blue); L chain (light blue) β-sheets; and the interface between the Nb (grey) and COP-1 L chain (light blue). (c) Local resolution estimates of the 2.6 Å hsCLDN-4/cCpE/COP-1/Nb “loose” masked map. Regions of the map are colored according to highest (blue) and lowest (red) resolutions.

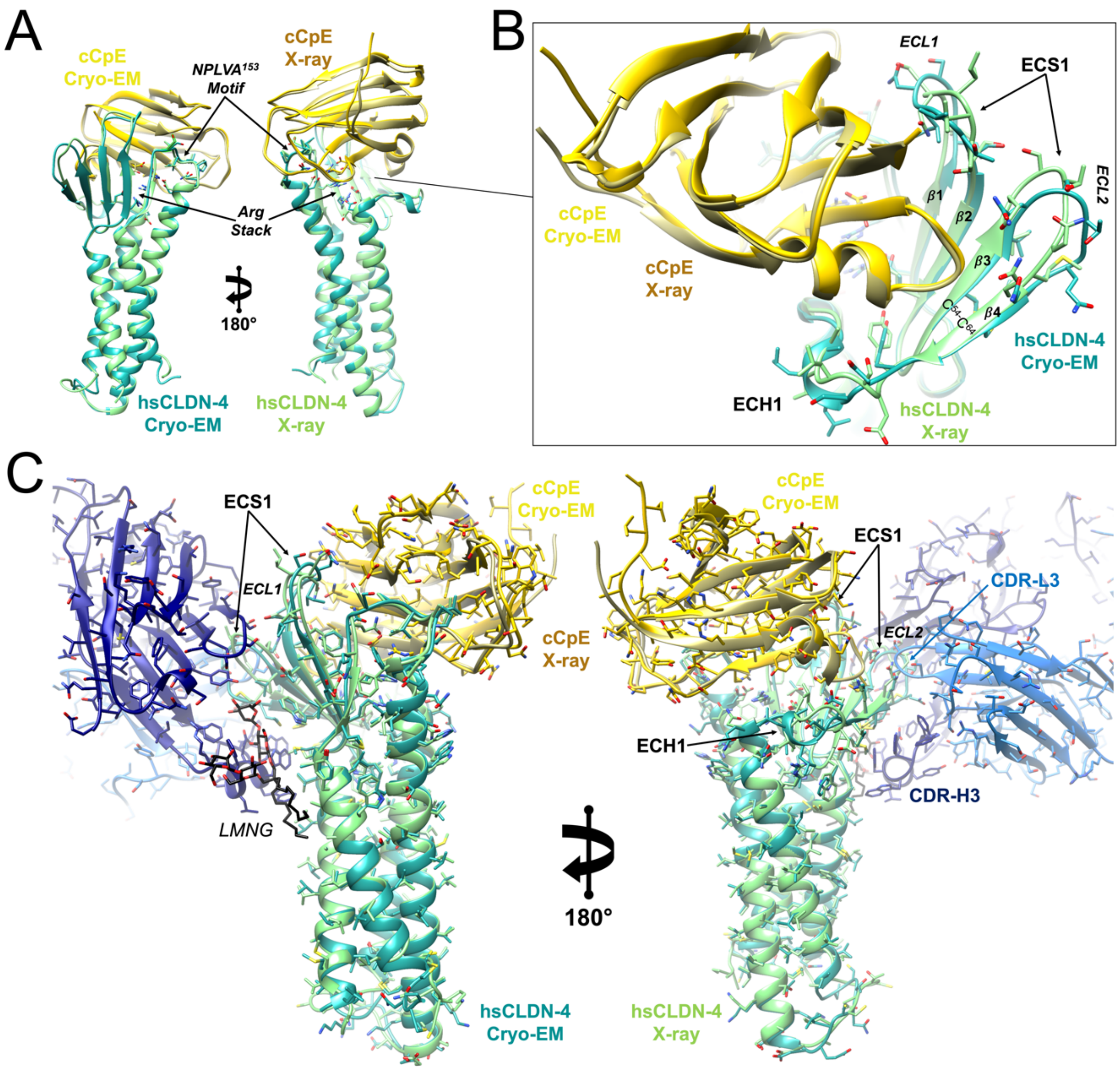

Supplementary Fig. 4. Structural Alignment of the hsCLDN-4/cCpE Complexes from Cryo-EM and X-ray Crystallography. (a) Overall alignment of the hsCLDN-4/cCpE complex from Cryo-EM (teal/gold) and X-ray (light green/copper). (b) Zoom-in of A depicting the structural differences between two extracellular loops (ECL) within ECS1 between the cryo-EM and X-ray structures. (c) Structural overlays of the cryo-EM and X-ray structures with COP-1 (blue) bound and shown for reference. Proteins are colored as in A,

Supplementary Table 1. Side Chains of Significance Used in hsCLDN-4 and COP-1 Interactions. Side chains that interact according to structural analyses are shown below. An interaction is defined as a polar or non-polar interaction that occurs between two atoms in these residues at a distance range between 1.4-4.0 Å.

COP-1		hsCLDN-4	
**CDR**	**H Chain**	**Epitope**	**Residues**
H1	Tyr59	1	Leu23
	His61		Ala26
H2	Ile77		Leu27
	Ser83		Pro28
	Ser85		Met29
H3	Trp130	2	Ser43
	Trp131	3	Gln57
	Trp132		Ser58
			Thr59
			Gly60
			Met62
			Cys64

COP-1		hsCLDN-4	
**CDR**	**L Chain**	**Epitope**	**Residues**
L1	Ser56	3	Gln57
L3	Ser116		Ser58
	Ser117		Thr59
	Ser118		Gln61
	Ser119		Gln63
	Ile121		

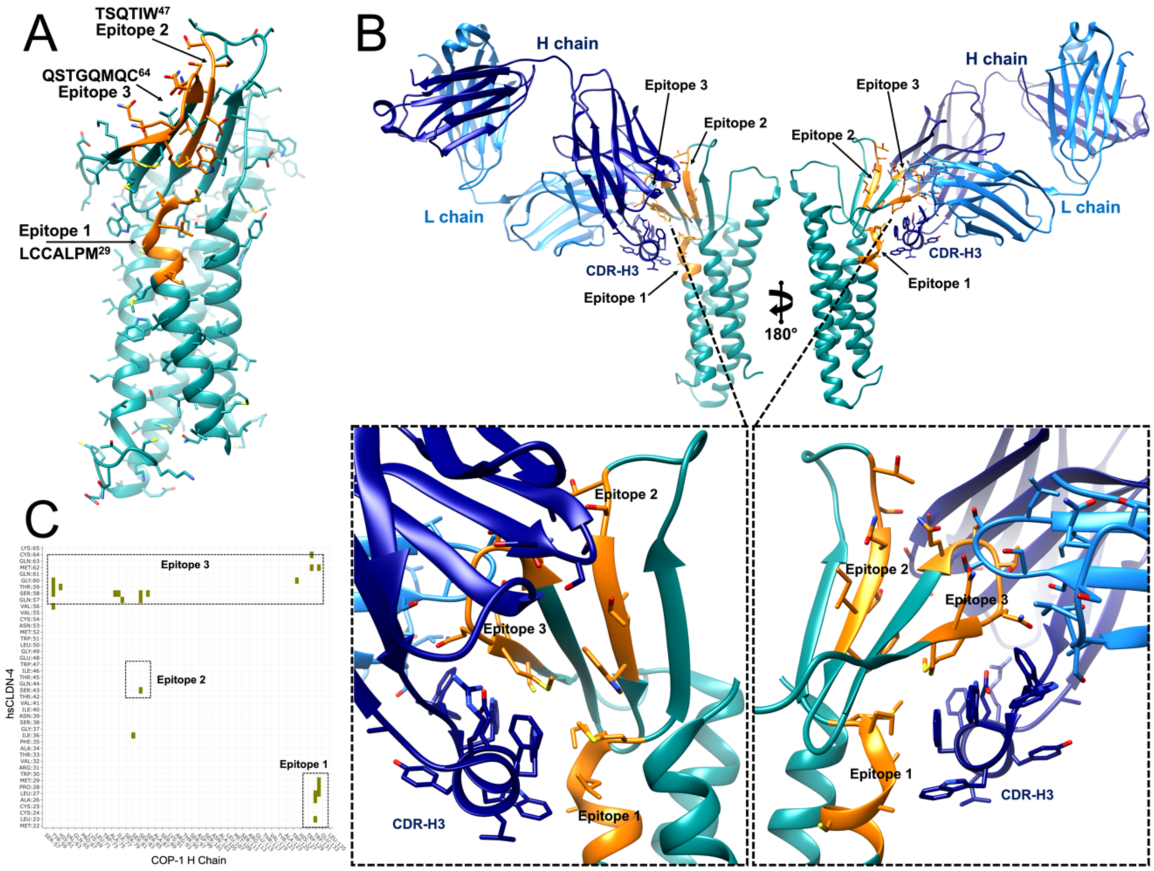

Supplementary Fig. 5. COP-1 Binding Epitopes on hsCLDN-4. (a) The three epitopes (orange) and their associated sequences on hsCLDN-4 (teal) that COP-1 uses to recognize it are labeled and shown with side chains represented as sticks. Epitope 1 constitutes residues LCCALPM^{29}; epitope 2, TSQTIW^{47}; and epitope 3, QSTGQMQC^{64} of hsCLDN-4, respectively. (b) hsCLDN-4 is colored as in A with COP-1 H (dark blue) or L chains (light blue) shown for reference. Various CDRs of COP-1 can be seen interacting with hsCLDN-4 epitopes 1, 2, and 3. (c) 2D contact plot of hsCLDN-4 and COP-1 H chain from MAPIYA [1].

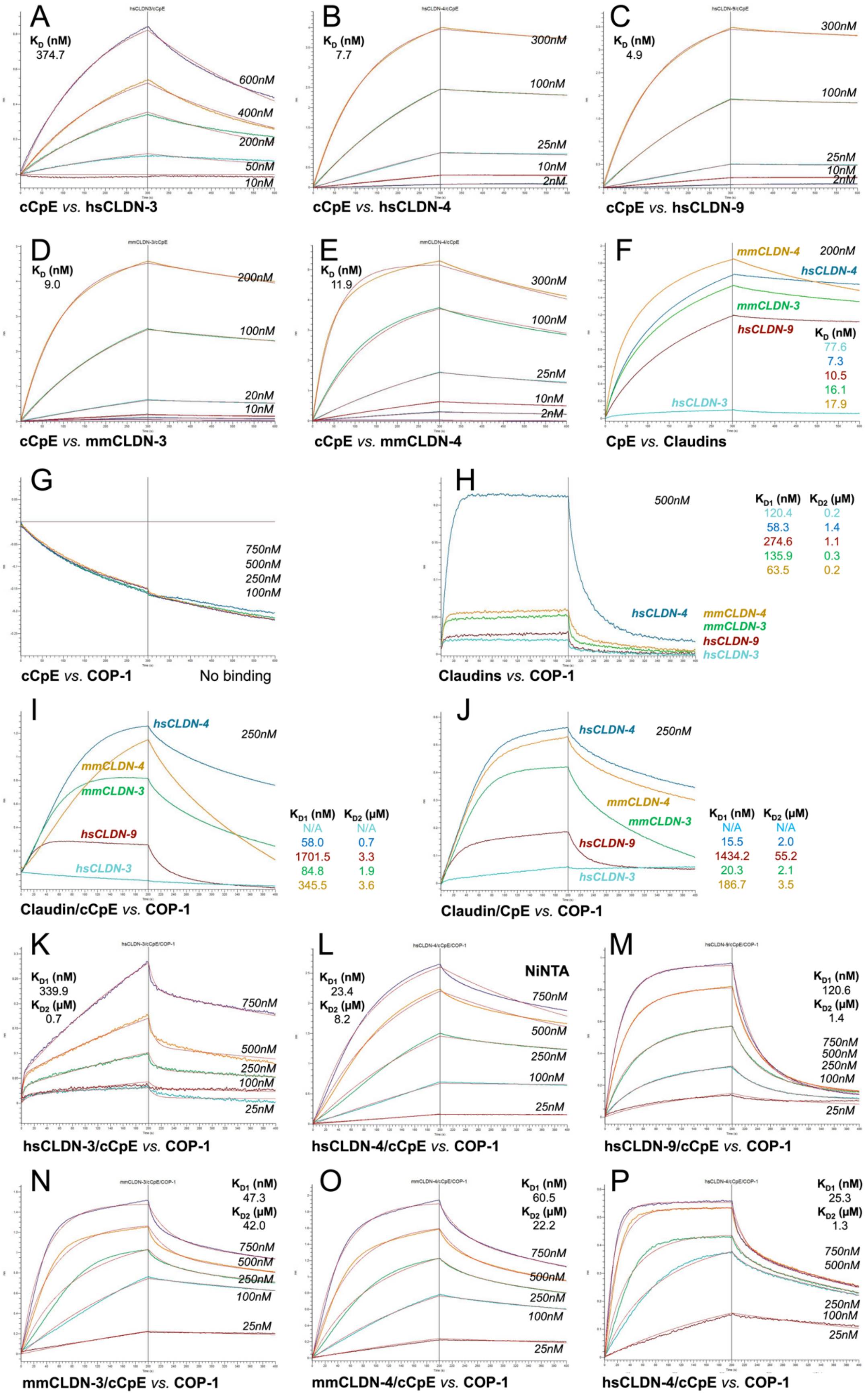
A
K_D (nM)
374.7
600nM
400nM
200nM
50nM
10nM
cCpE vs. hsCLDN-3
B
K_D (nM)
7.7
300nM
100nM
25nM
10nM
2nM
cCpE vs. hsCLDN-4
C
K_D (nM)
4.9
300nM
100nM
25nM
10nM
2nM
cCpE vs. hsCLDN-9
D
K_D (nM)
9.0
200nM
100nM
20nM
10nM
cCpE vs. mmCLDN-3
E
K_D (nM)
11.9
300nM
100nM
25nM
10nM
2nM
cCpE vs. mmCLDN-4
F
mmCLDN-4
200nM
hsCLDN-4
mmCLDN-3
hsCLDN-9
K_D (nM)
77.6
7.3
10.5
16.1
17.9
hsCLDN-3
CpE vs. Claudins
G
750nM
500nM
250nM
100nM
cCpE vs. COP-1
No binding
H
500nM
K_{D1} (nM) K_{D2} (μM)
120.4 0.2
58.3 1.4
274.6 1.1
135.9 0.3
63.5 0.2
hsCLDN-4
mmCLDN-4
mmCLDN-3
hsCLDN-9
hsCLDN-3
Claudins vs. COP-1
I
hsCLDN-4
250nM
mmCLDN-4
mmCLDN-3
hsCLDN-9
hsCLDN-3
K_{D1} (nM) K_{D2} (μM)
N/A N/A
58.0 0.7
1701.5 3.3
84.8 1.9
345.5 3.6
Claudin/cCpE vs. COP-1
J
hsCLDN-4
250nM
mmCLDN-4
mmCLDN-3
hsCLDN-9
hsCLDN-3
K_{D1} (nM) K_{D2} (μM)
N/A N/A
15.5 2.0
1434.2 55.2
20.3 2.1
186.7 3.5
Claudin/CpE vs. COP-1
K
K_{D1} (nM)
339.9
K_{D2} (μM)
0.7
750nM
500nM
250nM
100nM
25nM
hsCLDN-3/cCpE vs. COP-1
L
K_{D1} (nM)
23.4
K_{D2} (μM)
8.2
NiNTA
750nM
500nM
250nM
100nM
25nM
hsCLDN-4/cCpE vs. COP-1
M
K_{D1} (nM)
120.6
K_{D2} (μM)
1.4
750nM
500nM
250nM
100nM
25nM
hsCLDN-9/cCpE vs. COP-1
N
K_{D1} (nM)
47.3
K_{D2} (μM)
42.0
750nM
500nM
250nM
100nM
25nM
mmCLDN-3/cCpE vs. COP-1
O
K_{D1} (nM)
60.5
K_{D2} (μM)
22.2
750nM
500nM
250nM
100nM
25nM
mmCLDN-4/cCpE vs. COP-1
P
K_{D1} (nM)
25.3
K_{D2} (μM)
1.3
750nM
500nM
250nM
100nM
25nM
hsCLDN-4/cCpE vs. COP-1

Supplementary Fig. 6. BLI Sensograms Depicting Binding of Various Protein Complexes. Sensograms are grouped accordingly: a-f, Full and semi-quantitative experiments of claudin binding to enterotoxins in the absence of COP-1; g-j, Qualitative binding of COP-1 to cCpE, claudins, and claudin/enterotoxin complexes; and k-p, Quantitative kinetic binding experiments of COP-1 binding to claudin/cCpE complexes. Specifically, $cCpE_{-His10}$ immobilized on NiNTA sensors bound to (a) human claudin-3, (b) human claudin-4, (c) human claudin-9, (d) mouse claudin-3, and (e) mouse claudin-4. (f) shows CpE_{-His10} immobilized on NiNTA sensors binding against 200 nM of aforementioned claudins. Inset table reports the estimated K_D from these single-point analyses. All claudin/enterotoxin binding results (a-f) were fit using a 1:1 binding model. (g) shows $cCpE_{-His10}$ immobilized on NiNTA sensors binding against 100-750 nM COP-1 without claudins. (h) shows $claudin_{-Biotin}$ immobilized on SA sensors binding against 500 nM COP-1 without cCpE. Inset table reports the estimated $K_{D's}$ from these single-point analyses. (i) shows $cCpE_{-His10}$/claudin complexes immobilized on NiNTA sensors binding against 250 nM COP-1. Inset table reports the estimated $K_{D's}$ from these single-point analyses. (j) shows CpE_{-His10}/claudin complexes immobilized on NiNTA sensors binding against 250 nM COP-1 and inset table reports the estimated $K_{D's}$ from these single-point analyses. $cCpE_{-His10}$/human claudin-3 (k), $cCpE_{-His10}$/human claudin-4 (l), $cCpE_{-His10}$/human claudin-9 (m), $cCpE_{-His10}$/mouse claudin-3 (n), and $cCpE_{-His10}$/mouse claudin-4 (o) complexes immobilized on NiNTA sensors bound to COP-1. (p) shows $cCpE_{-His10}$/human claudin-$4_{-Biotin}$ immobilized on SA sensors bound to COP-1. All COP-1 binding results (h-p) were fit using a 2:1 heterogenous ligand binding model. Methodological details of this experiment appear in **Supplementary Table 5**.

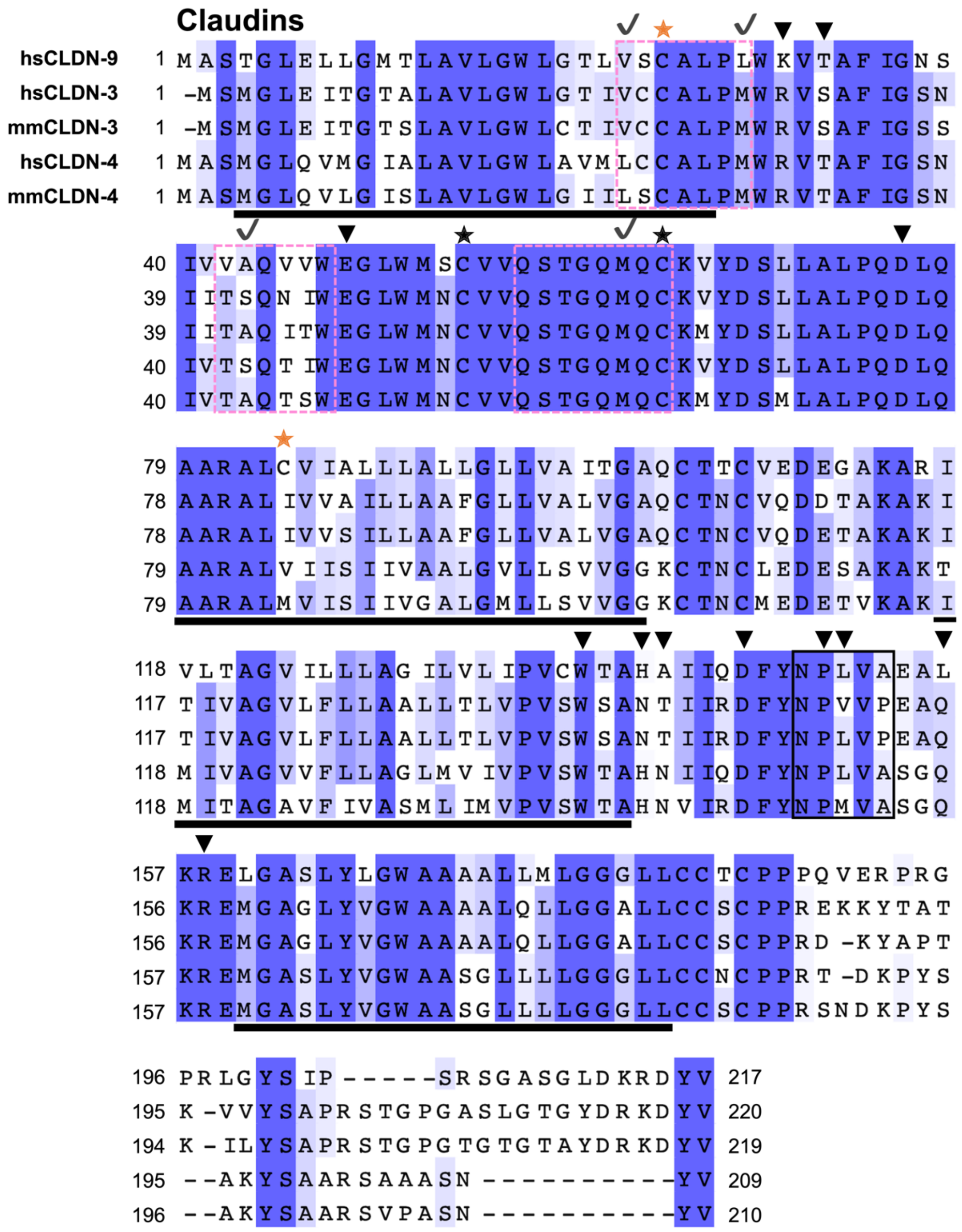

Supplementary Fig. 7. Sequence Alignment of Claudins. The sequences of claudins used in this study were aligned. Highlighted within the sequences are the following: ★ (black) represent the conserved disulfide bond in ECS1; ★ (orange) represent the disulfide bond in TM1-TM2 in hsCLDN-9; ▼(black) depict residues that constitute the cCpE-binding motif; box (black) highlights the NPLVA[153] motif; and ✓(black) shows residues predicted to drive COP-1 interactions with hsCLDN-4. Dashed boxes (pink) denote COP-1 interaction epitopes 1-3 present on claudins. Underlined bars (black) show the hydrophobic residues that comprise TM1-4 in claudins.

Supplementary Table 2. BLI Binding Results for Multi-Point Analyses with Kinetics

Table 1 and Supplementary Fig. 6a-e Data

Complex	k_{on} (1/Ms)	k_{off} (1/s)	K_D (nM)	$t_{1/2}$ (min)
hsCLDN-3 / cCpE	6.0×10^{3}	2.3×10^{-3}	374.7 ± 2.1	5.0
hsCLDN-4 / cCpE	2.7×10^{4}	2.1×10^{-4}	7.7 ± 0.1	55.0
hsCLDN-9 / cCpE	2.7×10^{4}	1.3×10^{-4}	4.9 ± 0.1	88.9
mmCLDN-3 / cCpE	5.0×10^{4}	4.5×10^{-4}	9.0 ± 0.1	25.7
mmCLDN-4 / cCpE	6.9×10^{4}	8.2×10^{-4}	11.9 ± 0.1	14.1

Table 1 and Supplementary Fig. 6k-p Data

Complex	$k_{on\ 1}$ (1/Ms)	$k_{off\ 1}$ (1/s)	$K_{D\ 1}$ (nM)	$k_{on\ 2}$ (1/Ms)	$k_{off\ 2}$ (1/s)	$K_{D\ 2}$ (μM)	$t_{1/2}$ (min)
hsCLDN-3•cCpE / COP-1	2.2×10^{5}	7.9×10^{-2}	339.9 ± 9.4	7.6×10^{4}	6.1×10^{-2}	0.7 ± 1.5	0.1
hsCLDN-4•cCpE / COP-1	2.4×10^{4}	5.5×10^{-4}	23.4 ± 1.7	2.6×10^{3}	2.1×10^{-2}	8.2 ± 6.6	21.2
hsCLDN-9•cCpE / COP-1	3.6×10^{4}	4.9×10^{-3}	120.6 ± 0.8	2.3×10^{4}	3.3×10^{-2}	1.4 ± 0.1	2.4
mmCLDN-3•cCpE / COP-1	2.7×10^{4}	1.1×10^{-3}	47.3 ± 1.0	0.7×10^{3}	2.8×10^{-2}	42.0 ± 24.9	10.6
mmCLDN-4•cCpE / COP-1	1.9×10^{4}	9.7×10^{-7}	60.5 ± 2.1	1.0×10^{3}	1.9×10^{-2}	22.2 ± 4.0	11.9
hsCLDN-4•cCpE / COP-1 (SA)	8.3×10^{4}	2.1×10^{-3}	25.3 ± 0.2	4.0×10^{4}	5.3×10^{-2}	1.3 ± 0.1	5.5

Binding data from each complex represent a single experiment.

Supplementary Table 3. BLI Binding Results for Single-Point Analyses

Supplementary Fig. 6f Data

Complex	K_D (nM)
hsCLDN-3 / CpE	77.6 ± 0.9
hsCLDN-4 / CpE	7.3 ± 0.2
hsCLDN-9 / CpE	10.5 ± 0.2
mmCLDN-3 / CpE	16.1 ± 0.2
mmCLDN-4 / CpE	17.9 ± 0.2

Supplementary Fig. 6h Data

Complex	$K_{D\,1}$ (nM)	$K_{D\,2}$ (μM)
hsCLDN-3 / COP-1	120.4 ± 44.3	0.2 ± 0.1
hsCLDN-4 / COP-1	58.3 ± 24.7	1.4 ± 3.6
hsCLDN-9 / COP-1	274.6 ± 15.8	1.1 ± 0.5
mmCLDN-3 / COP-1	135.9 ± 5.8	0.3 ± 0.1
mmCLDN-4 / COP-1	63.5 ± 1.5	0.2 ± 0.1

Supplementary Fig. 6i Data

Complex	$K_{D\,1}$ (nM)	$K_{D\,2}$ (μM)
hsCLDN-3•cCpE / COP-1	*N/A*	*N/A*
hsCLDN-4•cCpE / COP-1	58.0 ± 8.5	0.7 ± 1.0
hsCLDN-9•cCpE / COP-1	1701.5 ± 150.9	3.3 ± 2.4
mmCLDN-3•cCpE / COP-1	84.8 ± 6.7	1.9 ± 1.2
mmCLDN-4•cCpE / COP-1	345.5 ± 60.5	3.6 ± 3.3

Supplementary Fig. 6j Data

Complex	$K_{D\,1}$ (nM)	$K_{D\,2}$ (μM)
hsCLDN-3•CpE / COP-1	*N/A*	*N/A*
hsCLDN-4•CpE / COP-1	15.5 ± 3.6	2.0 ± 4.2
hsCLDN-9•CpE / COP-1	1434.2 ± 67.4	55.2 ± 37.6
mmCLDN-3•CpE / COP-1	20.3 ± 3.7	2.1 ± 3.7
mmCLDN-4•CpE / COP-1	186.7 ± 58.3	3.5 ± 21.6

Supplementary Fig. 9 Data

Complex	$K_{D\,1}$ (nM)	$K_{D\,2}$ (μM)
hsCLDN-4 / cCpE	7.3 ± 0.1	*N/A*
hsCLDN-4^{W47A} / cCpE	5.2 ± 0.1	*N/A*
hsCLDN-4$^{TSQT\text{-}AAAA45}$ / cCpE	47.5 ± 0.3	*N/A*
hsCLDN-9 / cCpE	5.2 ± 0.1	*N/A*
hsCLDN-9^{L29M} / cCpE	4.8 ± 0.1	*N/A*
hsCLDN-9$^{VAQVV\text{-}TSQTI46}$ / cCpE	3.7 ± 0.1	*N/A*
hsCLDN-4^{Amphipol} / cCpE	42.9 ± 0.2	*N/A*
hsCLDN-4^{NanodiscSoyPolar} / cCpE	4.1 ± 0.1	*N/A*
hsCLDN-4^{NanodiscEggPC} / cCpE	12.7 ± 0.3	*N/A*
hsCLDN-4•cCpE / COP-1	19.5 ± 2.4	7.4 ± 1.8
hsCLDN-4^{W47A}•cCpE / COP-1	152.3 ± 25.2	5.2 ± 6.1
hsCLDN-4$^{TSQT\text{-}AAAA45}$•cCpE / COP-1	380.2 ± 69.4	2.1 ± 2.1
hsCLDN-9•cCpE / COP-1	442.7 ± 203.2	9.7 ± 1.5
hsCLDN-9^{L29M}•cCpE / COP-1	21.6 ± 1.7	5.7 ± 1.2
hsCLDN-9$^{VAQVV\text{-}TSQTI46}$•cCpE / COP-1	3500.4 ± 1225.2	3.7 ± 2.8
hsCLDN-4^{Amphipol}•cCpE / COP-1	54.5 ± 0.5	0.1 ± 0.1
hsCLDN-4^{NanodiscSoyPolar}•cCpE / COP-1	69.2 ± 0.5	4.6 ± 5.3
hsCLDN-4^{NanodiscEggPC}•cCpE / COP-1	43.0 ± 3.1	4.0 ± 1.5

N/A = Not Applicable; *Binding data from each complex represent a single experiment.*

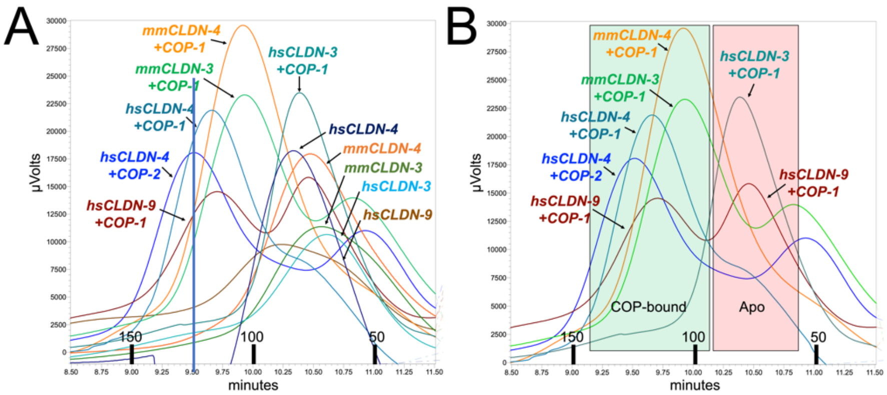

C

Sample Injected	Retention Time (min)	Complex?
hsCLDN-3/cCpE	10.60	No
hsCLDN-4/cCpE	10.35	No
hsCLDN-9/cCpE	10.25	No
mmCLDN-3/cCpE	10.55	No
mmCLDN-4/cCpE	10.45	No
hsCLDN-3/cCpE/COP-1	10.40	No
hsCLDN-4/cCpE/COP-1	9.65	Yes
hsCLDN-9/cCpE/COP-1	9.70/10.45	Yes/No
mmCLDN-3/cCpE/COP-1	9.95	Yes
mmCLDN-4/cCpE/COP-1	9.90	Yes
hsCLDN-4/cCpE/COP-2	9.50	Yes

Supplementary Fig. 8. Biochemical Validation of COP-1 Biophysical Findings. Complexes of claudins and cCpE$_{\text{-His10}}$ from BLI 96-well plates were injected alone or incubated with excess COP-1, then each sample was injected onto a SEC column equilibrated in BLI buffer. Decreases in peak retention times correlate to increased molecular masses as a result of COP-1 binding to claudin/cCpE complexes. Black bars on the x-axis represent approximate MWs of complexes in kDa. (a) SEC peaks of hsCLDN-3/cCpE (light blue), hsCLDN-4/cCpE (dark blue), hsCLDN-9/cCpE (brown), mmCLDN-3/cCpE (green), and mmCLDN-4/cCpE (orange) from BLI. Overlaid are SEC peaks from BLI incubated with COP-1of hsCLDN-3/cCpE (light teal), hsCLDN-4/cCpE (dark teal), hsCLDN-9/cCpE (maroon), mmCLDN-3/cCpE (light green), and mmCLDN-4/cCpE (light orange). The control sample of hsCLDN-4/cCpE bound to COP-2 (blue) is shown to validate that the peaks eluting <10 minutes represent sFab-bound complexes. (b) Data from incubations with only excess COPs from the same group of experiments are shown and colored as in A. Boxes representing COP-bound (green) or unbound (red) are added to clarify the two states. (c) Accessory table reporting peak retention times of the various samples to measure complex formation. Complex is defined as COP-1 bound or unbound.

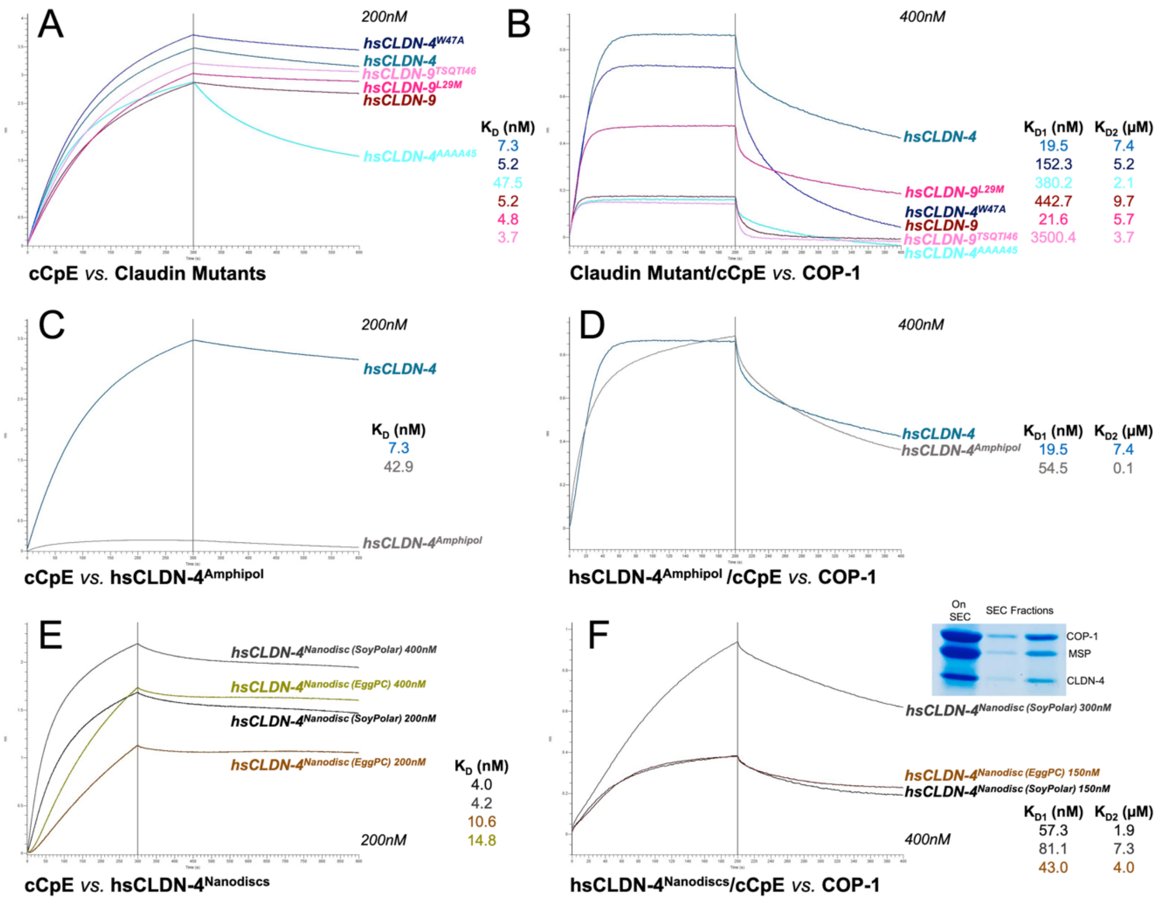

Supplementary Fig. 9. BLI Sensograms of Claudin Mutant and Non-Detergent Claudin Binding to cCpE and COP-1. Binding experiments consisted of single-point qualitative analyses in BLI buffer with DDM for detergent claudins or without DDM for non-detergent claudin analyses. Sensograms are grouped accordingly: (a) Claudin mutant (200 nM) binding to cCpE in the absence of COP-1 in DDM; (b) COP-1 (400 nM) binding to claudin mutant/cCpE complexes in DDM; (c), hsCLDN-4 in amphipol (200 nM) binding to cCpE without detergent; (d) COP-1 (400 nM) binding to hsCLDN-4 in amphipol/cCpE complexes without detergent; (e) hsCLDN-4 in 1D1 nanodiscs composed of soy polar lipids or egg PC lipid (200 and 400 nM) binding to cCpE without detergent; and (f) COP-1 (400 nM) binding to hsCLDN-4 in 1D1 nanodiscs composed of soy polar lipids or egg PC lipid binding/cCpE complexes without detergent. Inset shows representative SDS-PAGE gel of proteins reconstituted in nanodiscs. In c, hsCLDN-4^{Amphipol} signal is shown compared to hsCLDN-4 in DDM. In e and f, two concentrations of hsCLDN-4^{Nanodisc} were used to validate concentration-dependent binding signal. hsCLDN-4^{Amphipol} and hsCLDN-4^{Nanodisc} binding to unloaded NiNTA sensors showed no non-specific binding, and COP-1 also exhibited no significant binding signal to unloaded NiNTA sensors or cCpE-loaded sensors with no claudin in absence of detergent at 400 nM. All cCpE binding results (a, c, and e) were fit using a 1:1 binding model while all COP-1 results (b, d, and f) were fit using a 2:1 heterogenous ligand binding model. For the latter, two K_{Ds} are reported.

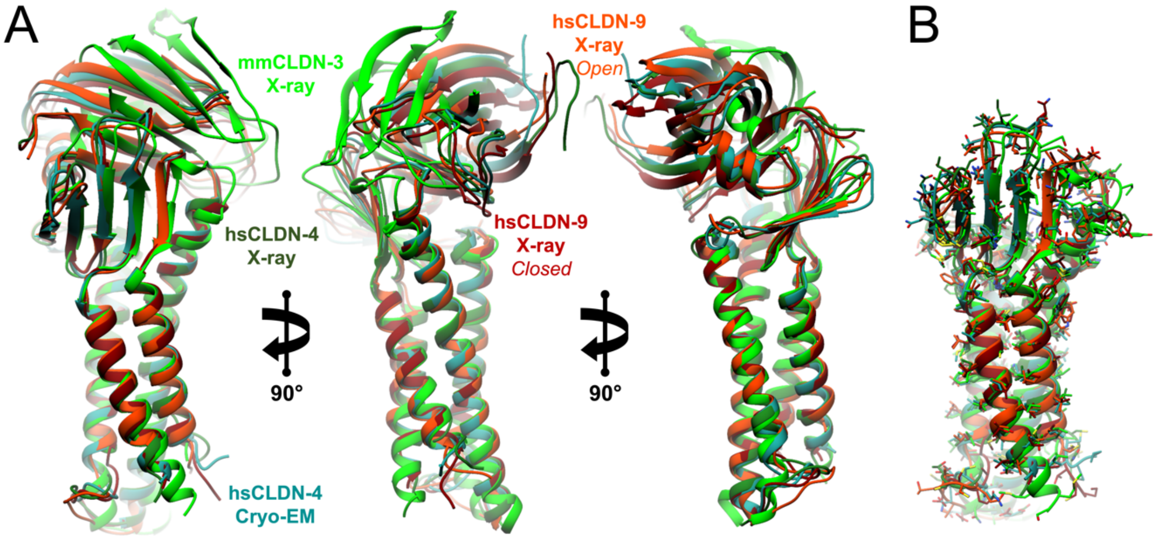

Supplementary Fig. 10. Conformation Comparison between Claudin/cCpE Complexes. Structures of claudins in complex with cCpE as determined by X-ray crystallography and cryo-EM were structurally superposed. (a) hsCLDN-4/cCpE (teal) from cryo-EM (this study) *versus* PDB IDs: 7KP4 hsCLDN-4/cCpE (green), 6AKE mmCLDN-3/cCpE (light green), and 6OV2 and 6OV3 hsCLDN-9/cCpE (red and orange) from X-ray crystallography are shown and rotated 90° to show three orientations. (b) Claudin complexes as shown in A but with cCpE is removed from all five structures to reveal claudin-specific movements. This analysis shows the minimal alterations that occur in the global structure of claudins.

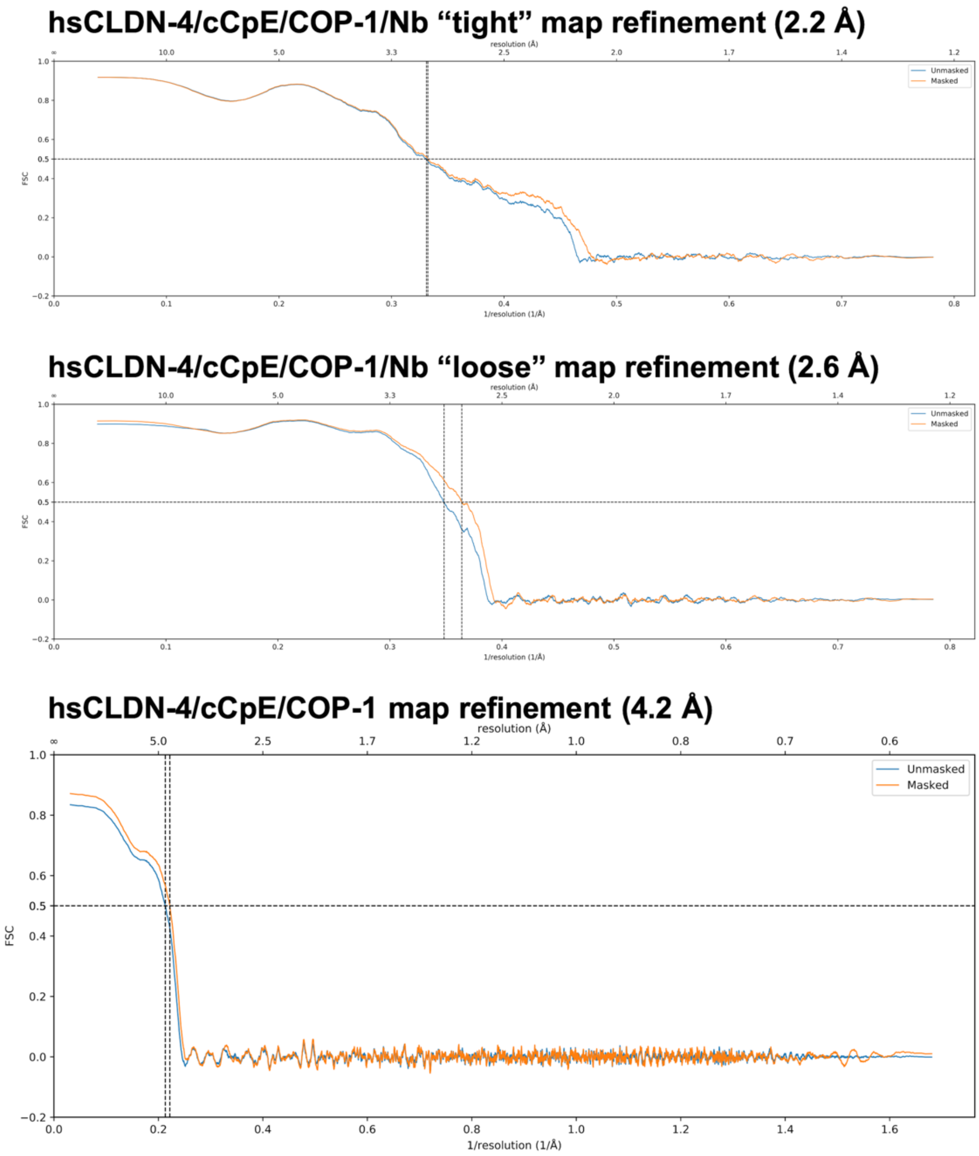

Supplementary Fig. 11. Cryo-EM Model to Map Validations. Map versus Model FSC curves generated by Phenix real-space refinement of the three determined structures. Intersections of curves at FSC = 0.5 are shown.

Supplementary Table 4. Experimental Design of BLI Binding Studies. BLI experimental design explaining the sensor type, immobilized ligand, protein analyte, analysis type (single-point qualitative or multi-point quantitative), and where associated experimental binding curves are presented in **Supplementary Figs. 6** and **9**.

Sensor	Ligand(s)	Analyte(s)	Analysis	Figure(s)
NiNTA	cCpE-$_{His10}$	claudin-$_{Biotin}$	Multi-point	S6a-e
NiNTA	CpE-$_{His10}$	claudin-$_{Biotin}$	Single-point	S6f
NiNTA	cCpE-$_{His10}$	COP-1	Single-point	S6g
SA	claudin-$_{Biotin}$	COP-1	Single-point	S6h
NiNTA	claudin-$_{Biotin}$/cCpE-$_{His10}$ complexes	COP-1	Single-point	S6i
NiNTA	claudin-$_{Biotin}$/CpE-$_{His10}$ complexes	COP-1	Single-point	S6j
NiNTA	claudin-$_{Biotin}$/cCpE-$_{His10}$ complexes	COP-1	Multi-point	S6k-o
SA	hsCLDN-4-$_{Biotin}$/cCpE-$_{His10}$ complexes	COP-1	Multi-point	S6p
NiNTA	cCpE-$_{His10}$	^{mutant}claudin	Single-point	S9a
NiNTA	^{mutant}claudin/cCpE-$_{His10}$ complexes	COP-1	Single-point	S9b
NiNTA	cCpE-$_{His10}$	$^{non\text{-}detergent}$claudin	Single-point	S9c and e
NiNTA	$^{non\text{-}detergent}$claudin/cCpE-$_{His10}$ complexes	COP-1	Single-point	S9d and f

Supplementary References

1. Badaczewska-Dawid, A.E., Nithin, C., Wroblewski, K., Kurcinski, M. & Kmiecik, S. MAPIYA contact map server for identification and visualization of molecular interactions in proteins and biological complexes. *Nucleic Acids Res* **50**, W474-W482 (2022).
